# Genetic Subtypes and Outcome of Patients Aged 1 to 45 Years Old With Acute Lymphoblastic Leukemia in the NOPHO ALL2008 Trial

**DOI:** 10.1097/HS9.0000000000000883

**Published:** 2023-05-04

**Authors:** Ulrika Norén-Nyström, Mette K. Andersen, Gisela Barbany, Vaidas Dirse, Martine Eilert-Olsen, Marie Engvall, Arja Harila-Saari, Mats Heyman, Randi Hovland, Satu Häikiö, Jón J. Jónsson, Ritva Karhu, Eigil Kjeldsen, Anna Norberg, Birgitte S. Preiss, Kati Pulkkinen, Petter Quist-Paulsen, Hannele Räsänen, Kjeld Schmiegelow, Anne Seitsonen, Helene Sjögren, Pille Tammur, Bertil Johansson

**Affiliations:** 1 Department of Clinical Sciences, Pediatrics, Umeå University, Sweden; 2 Department of Clinical Genetics, University Hospital Rigshospitalet, Copenhagen, Denmark; 3 Department of Molecular Medicine and Surgery, Karolinska Institutet, and Clinical Genetics, Karolinska University Hospital, Stockholm, Sweden; 4 Hematology, Oncology and Transfusion Medicine Center, Vilnius University Hospital Santaros Klinikos, Lithuania; 5 Section for Cancer Cytogenetics, Institute for Cancer Genetics and Informatics, The Norwegian Radium Hospital, Oslo University Hospital, Norway; 6 Department of Immunology, Genetics and Pathology, Uppsala University, Sweden; 7 Department of Women’s and Children’s Health, Uppsala University, Sweden; 8 Childhood Cancer Research Unit, Department of Women’s and Children’s Health, Karolinska Institutet, Stockholm, Sweden; 9 Department of Paediatric Oncology, Karolinska University Hospital, Stockholm, Sweden; 10 Department for Medical Genetics, Haukeland University Hospital, Bergen, Norway; 11 Department of Genomics, Laboratory Division, Turku University Hospital, Finland; 12 Department of Genetics and Molecular Medicine, Landspitali, Reykjavik, Iceland; 13 Department of Biochemistry and Molecular Biology, Faculty of Medicine, University of Iceland, Reykjavik, Iceland; 14 Laboratory of Clinical Genetics, Fimlab Laboratories, Tampere, Finland; 15 Department of Hematology, Cancer Cytogenetics Section, Aarhus University Hospital, Denmark; 16 Department of Medical Biosciences, Medical and Clinical Genetics, Umeå University, Sweden; 17 Department of Pathology, Odense University Hospital, Denmark; 18 Laboratory of Genetics, Eastern Finland Laboratory Centre, Kuopio, Finland; 19 Department of Hematology, St. Olav’s Hospital, Trondheim University Hospital, Norway; 20 Nordlab Oulu Genetics laboratory, Oulu, Finland; 21 Department of Pediatrics and Adolescent Medicine, University Hospital Rigshospitalet, Copenhagen, Denmark; 22 HUSLAB Laboratory of Genetics, University of Helsinki and Helsinki University Hospital, Finland; 23 Department of Clinical Genetics and Genomics, Sahlgrenska University Hospital, Gothenburg, Sweden; 24 Department of Clinical Genetics, Tartu University Hospital, Estonia; 25 Division of Clinical Genetics, Department of Laboratory Medicine, Lund University, Sweden; 26 Department of Clinical Genetics, Pathology, and Molecular Diagnostics, Office for Medical Services, Region Skåne, Lund, Sweden

The outcome of pediatric B-cell precursor acute lymphoblastic leukemia (BCP ALL) has improved tremendously in the last decades, from being an invariably fatal disease until the 1960s to an overall survival rate of >90% in high-income countries.^1^ This remarkable success has been achieved by, for example, the implementation of risk stratification based on measurable residual disease (MRD) levels and cytogenetic subtypes.^2^ There is now also ample evidence that genetic findings are equally important in adolescents and younger adults.^3^ One of the objectives of the Nordic Society of Pediatric Hematology and Oncology (NOPHO) ALL2008 trial was to ascertain if the cure rate of adult ALL could be improved by diagnostic work-up and risk-adapted treatment according to a pediatric protocol.^4–6^ The trial comprised 1771 patients of age 1–45 years with *BCR*::*ABL1*-negative BCP (n = 1493; 84%) or T-cell (n = 278; 16%) ALL diagnosed in the Nordic countries, Estonia, and Lithuania between July 2008 and February 2016 (Suppl. Information and Suppl. Table S1). Here, we report the clinical implications of genetic subtypes in this trial, focusing on BCP ALL.

The patients were stratified at 3 time points (Suppl. Information). The first stratification dichotomized the patients based on the white blood cell counts and on BCP versus T-cell ALL. The second, at the end of induction, was primarily based on the MRD status and cytogenetic findings. *KMT2A* rearrangements (*KMT2A*-r), near-haploidy (NH), and low hypodiploidy (HoL) were grouped as high-risk (HR) abnormalities, whereas intrachromosomal amplification of chromosome 21 (iAMP21), dic(9;20)(p13;q11), and *TCF3*::*PBX1* were intermediate-risk (IR) aberrations. The third stratification was mainly based on the MRD levels on day 79, resulting in the final risk groups standard risk (SR), IR, and HR stratified to chemotherapy only, and HR intended for stem cell transplantation.

Of the 278 T-cell ALLs, 18% were genetically normal, 71% abnormal, and 11% uninformative; these did not differ with respect to clinical findings at diagnosis, risk stratification, or outcome (Suppl. Table S2), in line with previous studies.^7^ Importantly, however, the outcome of T-cell ALL clearly improved compared with the previous NOPHO ALL2000 trial^8,9^ and several other trials (Suppl. Table S3). Eight (2.9%) patients had *KMT2A*-r-positive T-cell ALL (Suppl. Table S4) and 7 of them are alive in complete remission 1 (CR1) (1 patient succumbed to treatment-related toxicity), suggesting that the prognosis of *KMT2A*-r-positive T-cell ALL may be relatively favorable. However, international collaborations are required to analyze a sufficiently large number of cases to be able to address the prognostic implications of *KMT2A*-r in T-cell ALL.

Of the 1493 BCP ALLs, 32% were high hyperdiploid (HeH), 28% B-other, 23% *ETV6*::*RUNX1*, 5.1% genetically unknown, 3.4% *KMT2A*-r, 3.3% *TCF3*::*PBX1*, 1.9% dic(9;20), 1.8% iAMP21, 1.0% HoL, and 0.5% NH (Table 1). Not surprisingly, the 5-year probabilities of disease-free survival (pDFS) and overall survival (pOS) varied significantly (*P* < 0.001) among these subtypes (Figure 1; details on the statistical analyses performed and the definitions of pOS, pDFS, and probabilities of cumulative incidence of relapse [pCIR] and event-free survival [pEFS] are given in Suppl. information).

**Table 1:** Clinical Features of the Genetic Subtypes in the 1493 BCP ALL Patients Included in the NOPHO ALL2008 Trial

Variable	HeH	B-other	*ETV6*::	*KMT2A*-r	*TCF3*::	dic(9;20)	iAMP21	HoL	NH	Unknown
	n = 471	n = 421	*RUNX1*	n = 51	*PBX1*	n = 29	n = 27	n = 15	n = 8	n = 76
	(32%)	(28%)	n = 346	(3.4%)	n = 49	(1.9%)	(1.8%)	(1.0%)	(0.5%)	(5.1%)
			(23%)		(3.3%)					
Median age (range)	4.1 (1.1–44)	11 (1.0–45)	3.9 (1.4–35)	9.6 (1.0–45)	7.2 (1.3–44)	2.7 (1.0–25)	10 (5.1–44)	14 (5.7–45)	3.9 (2.2–8.8)	14 (1.1–41)
1–9	402 (85%)	201 (48%)	326 (94%)	26 (51%)	33 (67%)	26 (90%)	13 (48%)	3 (20%)	8 (100%)	33 (43%)
10–17	48 (10%)	111 (26%)	17 (4.9%)	8 (16%)	9 (18%)	1 (3.4%)	12 (44%)	7 (47%)	0	14 (18%)
18–45	21 (4.5%)	109 (26%)	3 (0.9%)	17 (33%)	7 (14%)	2 (6.9%)	2 (7.4%)	5 (33%)	0	29 (38%)
Sex										
Female	226 (48%)	185 (44%)	161 (47%)	30 (59%)	26 (53%)	18 (62%)	14 (52%)	10 (67%)	2 (25%)	35 (46%)
Male	245 (52%)	236 (56%)	185 (53%)	21 (41%)	23 (47%)	11 (38%)	13 (48%)	5 (33%)	6 (75%)	41 (54%)
Median WBC count × 10^9^/L (range)	6 (0.7–330)	10 (0.4–414)	10 (1.0–515)	91 (2.0–1161)	15 (1.0–420)	35 (1.0–374)	9.6 (1.0–57)	6 (2.0–332)	83 (1.0–546)	5.6 (1.0–388)
<100	461 (98%)	381 (90%)	325 (94%)	27 (53%)	46 (94%)	22 (76%)	27 (100%)	14 (93%)	4 (50%)	71 (93%)
≥100	10 (2.1%)	40 (9.5%)	21 (6.1%)	24 (47%)	3 (6.1%)	7 (24%)	0	1 (6.7%)	4 (50%)	5 (6.6%)
Missing	0	0	0	0	0	0	0	0	0	0
CNS status										
CNS1	423 (90%)	382 (91%)	312 (90%)	37 (73%)	46 (94%)	17 (59%)	25 (93%)	14 (93%)	8 (100%)	68 (89%)
CNS2	36 (7.6%)	29 (6.9%)	25 (7.2%)	7 (14%)	3 (6.1%)	9 (31%)	2 (7.4%)	1 (6.7%)	0	3 (3.9%)
CNS3	12 (2.5%)	10 (2.4%)	7 (2.0%)	7 (14%)	0	3 (10%)	0	0	0	3 (3.9%)
Missing	0	0	2 (0.6%)	0	0	0	0	0	0	2 (2.6%)
Stratification day 0^*a*^										
Pred induction	461 (98%)	381 (90%)	340 (98%)	27 (53%)	46 (94%)	22 (76%)	27 (100%)	14 (93%)	4 (50%)	71 (93%)
Dexa induction	10 (2.1%)	40 (9.5%)	6 (1.7%)^*b*^	24 (47%)	3 (6.1%)	7 (24%)	0	1 (6.7%)	4 (50%)	5 (6.6%)
Stratification day 29										
SR	302 (64%)	220 (52%)	240 (69%)	0	0	0	0	0	0	0
IR	147 (31%)	135 (32%)	99 (29%)	0	48 (98%)	27 (93%)	24 (89%)	0	0	64 (84%)
HR-chemo	7 (1.5%)	21 (5.0%)	2 (0.6%)	47 (92%)	0	2 (6.9%)	0	14 (93%)	8 (100%)	5 (6.6%)
HR-SCT	9 (1.9%)	42 (10%)	4 (1.2%)	0	0	0	3 (11%)	1 (6.7%)	0	4 (5.3%)
Not stratified^*c*^	6 (1.3%)	3 (0.7%)	1 (0.3%)	4 (7.8%)	1 (2%)	0	0	0	0	3 (3.9%)
Final stratification										
SR	301 (64%)	220 (52%)	240 (69%)	0	0	0	0	0	0	0
IR	144 (31%)	120 (29%)	99 (29%)	0	47 (96%)	27 (93%)	23 (85%)	0	0	60 (79%)
HR-chemo	7 (1.5%)	17 (4.0%)	2 (0.6%)	44 (86%)	0	2 (6.9%)	0	14 (93%)	8 (100%)	5 (6.6%)
HR-SCT	13 (2.8%)	61 (14%)	4 (1.2%)	3 (5.9%)	1 (2.0%)	0	4 (15%)	1 (6.7%)	0	8 (11%)
Not stratified^*c*^	6 (1.3%)	3 (0.7%)	1 (0.3%)	4 (7.8%)	1 (2.0%)	0	0	0	0	3 (3.9%)
Deaths	19 (4.0%)	65 (15%)	12 (3.5%)	16 (31%)	1 (2.0%)	1 (3.4%)	4 (15%)	5 (33%)	4 (50%)	9 (12%)
Events	44 (9.3%)	99 (24%)	32 (9.2%)	18 (35%)	2 (4.1%)	5 (17%)	10 (37%)	7 (47%)	4 (50%)	20 (26%)
Induction death	4 (0.8%)	3 (0.7%)	1 (0.3%)	4 (7.8%)	0	0	0	0	0	2 (2.6%)
Resistant disease	0	0	0	0	0	0	0	0	0	0
DCR1	6 (1.3%)	13 (3.1%)	5 (1.4%)	4 (7.8%)	1 (2.0%)	1 (3.4%)	1 (3.7%)	0	3 (38%)	2 (2.6%)
Relapse	28 (5.9%)	82 (19%)	23 (6.6%)	9 (18%)	1 (2.0%)	4 (14%)	9 (33%)	5 (33%)	1 (13%)	16 (21%)
BM	14 (50%)	51 (62%)	11 (48%)	7 (78%)	0	3 (75%)	7 (78%)	5 (100%)	1 (100%)	9 (56%)
CNS	6 (21%)	8 (9.8%)	9 (39%)	0	1 (100%)	0	0	0	0	2 (13%)
Test	0	1 (1.2%)	0	0	0	0	0	0	0	2 (13%)
Other site	2 (7.1%)	3 (3.7%)	0	2 (22%)	0	0	0	0	0	0
BM + CNS	4 (14%)	11 (13%)	1 (4.3%)	0	0	1 (25%)	2 (22%)	0	0	0
BM + Test	0	3 (3.7%)	1 (4.3%)	0	0	0	0	0	0	1 (6.3%)
BM + other site	0	4 (4.9%)	0	0	0	0	0	0	0	2 (13%)
BM, CNS + Test	2 (7.1%)	1 (1.2%)	1 (4.3%)	0	0	0	0	0	0	0
SMN	6 (1.3%)	1 (0.2%)	3 (0.9%)	1 (2.0%)	0	0	0	2 (13%)	0	0
pCIR at 5 y ± SE	0.06 ± 0.01	0.19 ± 0.02	0.06 ± 0.01	0.19 ± 0.06	0.02 ± 0.02	0.14 ± 0.06	0.31 ± 0.09	0.27 ± 0.11	0.12 ± 0.12	0.18 ± 0.05
pDFS at 5 y ± SE	0.92 ± 0.01	0.78 ± 0.02	0.91 ± 0.02	0.70 ± 0.07	0.96 ± 0.03	0.83 ± 0.07	0.66 ± 0.09	0.60 ± 0.13	0.50 ± 0.18	0.79 ± 0.05
pEFS at 5 y ± SE	0.91 ± 0.02	0.77 ± 0.02	0.92 ± 0.02	0.65 ± 0.07	0.96 ± 0.03	0.83 ± 0.07	0.66 ± 0.09	0.60 ± 0.13	0.50 ± 0.18	0.77 ± 0.05
pOS at 5 y ± SE	0.97 ± 0.01	0.86 ± 0.02	0.97 ± 0.01	0.69 ± 0.07	0.98 ± 0.02	0.97 ± 0.03	0.92 ± 0.05	0.67 ± 0.12	0.50 ± 0.18	0.88 ± 0.04

^*a*^All patients received the same induction chemotherapy with either prednisolone or dexamethasone.

^*b*^After an amendment in 2009, *ETV6*::*RUNX1*-positive ALL cases with WBC counts ≥100 × 10^9^/L received prednisolone instead of dexamethasone; hence, only the 6 initial cases with hyperleukocytosis were stratified to Dexa induction.

^*c*^Comprises patients who died during induction, were lost to follow-up, abandoned protocol therapy before day 29, or had received such a modified therapy that they were considered outliers.

BCP ALL = B-cell precursor acute lymphoblastic leukemia; BM = bone marrow; CNS = central nervous system; CNS1 = no blasts on cytospin and no signs of CNS leukemia; CNS2 = >0 and <5 cells/μL cerebrospinal fluid that on cytospin were regarded to represent leukemic blasts but no other signs of CNS leukemia; CNS3 = ≥5 cells/μL cerebrospinal fluid that on cytospin were regarded to represent leukemic blasts and/or signs of CNS leukemia; DCR1 = death in first complete remission; Dexa = dexamethasone; HeH = high hyperdiploid (51–67 chromosomes); HoL = low hypodiploidy (30–39 chromosomes); HR-chemo = high-risk patients stratified to treatment with chemotherapy only; HR-SCT = high-risk patients stratified to stem cell transplantation; iAMP21 = intrachromosomal amplification of chromosome 21; IR = intermediate risk; *KMT2A*-r = *KMT2A* rearrangement; n = number of patients; NH = near-haploidy (24–29 chromosomes); pCIR = probability of cumulative incidence of relapse; pDFS = probability of disease-free survival; pEFS = probability of event-free survival; pOS = probability of overall survival; Pred = prednisolone; SE = standard error; SMN = secondary malignant neoplasm; SR = standard risk; Test = testicular; WBC = white blood cell.

**Figure 1.**
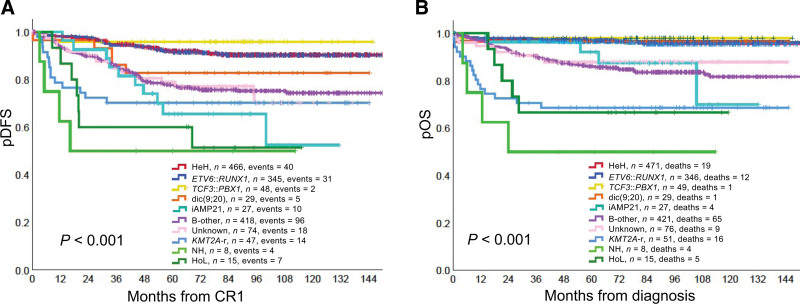
**The pDFS and pOS of the genetic B-cell precursor acute lymphoblastic leukemia subtypes**. (A) pDFS. (B) pOS. *CR1* = complete remission 1; *HeH* = high hyperdiploid (51–67 chromosomes); *HoL* = low hypodiploidy (30–39 chromosomes); *iAMP21* = intrachromosomal amplification of chromosome 21; *KMT2A-r* = rearrangement of the *KMT2A* gene; *NH* = near-haploidy (24–29 chromosomes); pDFS = probabilities of disease-free survival; pOS = probabilities of overall survival.

The pCIR, pDFS, pEFS, and pOS were particularly favorable for patients with HeH, *ETV6*::*RUNX1*, or *TCF3*::*PBX1* (Table 1, Figure 1, Suppl. Figures S1-S4, and Suppl. Table S5). These groups had 5-year pOS of ≥0.96; similar survival rates have been reported in recent treatment trials (Suppl. Tables S6-S8). Interestingly, the excellent outcome of the HeH and *ETV6*::*RUNX1* groups was achieved by SR chemotherapy in 67% of the patients. In fact, some of the patients may have been overtreated already by that treatment, as indicated by the nonnegligible frequency (~2.2%) of death in CR1 or second malignant neoplasm in the SR group (Suppl. Table S9).

Although dic(9;20)(p13;q11) does not result in a specific gene fusion and sometimes occur together with bona fide primary gene fusions, such as *BCR*::*ABL1*,^10,11^ it was considered a disease-defining/risk-stratifying abnormality in the NOPHO ALL2008 trial because it had been shown to be associated with a worse outcome than HeH and *ETV6*::*RUNX1* in the ALL2000 trial.^12^ Despite receiving intensified therapy, the pCIR, pDFS, and pEFS were disappointing; however, most relapses were salvageable, as exemplified by the pOS of 0.97 and by pOS ≥0.92 in a few other trials specifically addressing this aberration (Table 1 and Suppl. Table S10).

Considering the well-established dismal prognosis of iAMP21-positive ALL,^13^ this abnormality was risk-stratifying in the NOPHO ALL2008 trial. This notwithstanding, the pCIR, pDFS, and pEFS were unsatisfactory (Figure 1 and Table 1). Furthermore, although salvage therapy was surprisingly successful, resulting in 5-year pOS rates of 0.92 for both children and adolescents (Suppl. Table S11), both pDFS and pOS kept decreasing with longer follow-up (Suppl. Figure S2), clearly showing that the primary therapy for iAMP21-positive cases needs to be modified.

Because of the notoriously poor outcome of the *KMT2A*-r, HoL, and NH subtypes (Suppl. Tables S12-S14), these were all stratified as HR. The *KMT2A*-r-positive cases clearly benefited from this approach—the pEFS/pOS of children (0.77/0.81) and adolescents (0.75/0.88) were not only a substantial improvement compared with the previous ALL2000 trial but also superior to what has been reported by several other study groups (Suppl. Table S12). It is noteworthy that this excellent (for *KMT2A*-r) outcome of patients of age 1–17 years was achieved by HR chemotherapy alone, not SCT. In contrast, 4 of 8 patients with NH and 5 of 15 HoL patients died (Table 1); thus, improved treatment options are eagerly needed for both these subtypes.

In the multivariable analyses of the BCP ALL patients finally stratified to SR and IR (according to intention-to-treat; Suppl. Table S15), only age and genetic subtypes impacted prognosis, with age >17 years, iAMP21, B-other, and genetically uninformative having a significant negative prognostic impact on pDFS (Suppl. Table S16).

Age was clearly associated with both clinical and genetic features in the NOPHO ALL2008 trial. First, in T-cell ALL, the 5-year pOS decreased by age, being 0.82 for children aged 1–9 years, 0.75 for those aged 10–17 years, and 0.66 for adults (Suppl. Table S3); there was, however, no clear difference in the 5-year pOS between adults of age 18–29 years (0.67) and 30–45 years (0.64). Second, in BCP ALL, the frequencies of the cytogenetic and final risk stratification groups varied among the age groups. The proportions of HeH, *ETV6*::*RUNX1*, dic(9;20), NH, and SR decreased by age, whereas *KMT2A*-r, B-other, genetically unknown, IR, and HR increased by age (Suppl. Tables S17 and S18). Third, adults with *KMT2A*-r-positive BCP ALL had significantly inferior pCIR (0.40) and pOS (0.41) compared with those aged 1–9 (0.08/0.81) and 10–17 years (0.12/0.88) (*P* = 0.042 and 0.016, respectively; Suppl. Table S19). Unfortunately, SCT may not be the way forward, except perhaps for *KMT2A*::*AFF1*-positive cases that are MRD negative at the time of transplantation.^14^ Fourth, pDFS, pEFS, and pOS decreased and pCIR increased significantly by age for the patient with B-other ALL (*P* < 0.001 for all comparisons; Suppl. Table S19). In adult B-other, however, the differences in pDFS, pEFS, pOS, and pCIR between those aged 18–29 years and 30–45 years were not statistically significant (pDFS 0.65/0.57, pEFS 0.65/0.57, pOS 0.77/0.70, and pCIR 0.32/0.40; *P* > 0.1 for all comparisons). Fifth, the frequencies of genetically uninformative BCP ALLs increased by age, from 3% in children of age 1–9 years to 15% in adults (Suppl. Table S17). The “unknown” group had a relatively poor outcome (Table 1 and Figure 1), but its prognostic impact did differ among the age groups (Suppl. Table S19) or between adults of age 18–29 years and 30–45 years (pEFS 0.76/0.67, pDFS 0.76/0.67, pOS 0.90/0.62, and pCIR 0.30/0.33; *P* > 0.1 for all comparisons). The general dismal survival of this subtype is not unexpected considering that it most likely includes a mixture of cases with undetected abnormalities, some of which may well confer a poor prognosis. Although genetically uninformative cases obviously occur in all treatment trials, surprisingly little attention has been focused on this groups of patients. The unknown subtype should be investigated in detail and properly addressed in order to make sure that it is kept at a minimum in future treatment trials. Implementing various types of massive parallel sequencing, such as RNA and whole genome sequencing,^15^ should hopefully solve the problem because these methods ought to detect all relevant genetic abnormalities. Finally, with regard to age, it is noteworthy that the pDFS, pEFS, and pOS of patients with HeH ALL did not differ significantly among the age groups 1–9, 10–17, and 18–45 years (Suppl. Table S19). Interestingly, the favorable survival of adult HeH, compared to previous trials (Suppl. Table S6), was the result of SR or IR treatment in all cases except one (data not shown).

In conclusion, the survival rates increased for most of the genetic BCP ALL subtypes in the NOPHO ALL2008 trial compared with the previous ALL2000 trial. However, the treatment of older patients and of the subtypes iAMP21 and B-other need be improved, and so does the genetic characterization of the B-other and genetically uninformative groups. To increase survival further, development of targeted therapy for some of the subtypes is clearly needed.

## ACKNOWLEDGMENTS

We thank all participating patients and their parents/guardians. Methodological consultations were provided by Northern Register Center, Umeå University Hospital, Umeå, Sweden.

## AUTHOR CONTRIBUTIONS

UNN and BJ conceived and designed the study, made sure that the final genetic classification was correct, and wrote the article. AN, AS, BJ, BSP, GB, EK, HR, HS, JJJ, KP, ME, MEO, MKA, PT, RH, RK, SH, and VD provided and reviewed all genetic data. AHS, MH, PQP, and UNN supplied clinical data. KS was responsible for the overall design of the NOPHO ALL2008 protocol. All authors edited and approved the final version of the article.

## DISCLOSURES

The authors have no conflicts of interest to disclose.

## SOURCES OF FUNDING

The Swedish Childhood Cancer Foundation (PR2018-0004 and PR2021-0005, grant holder: BJ; KF2017-0010, PL2018-0007, and TJ2021-0143, grant holder: UNN), the Swedish Cancer Society (20 0792 PjF, grant holder: BJ), the Swedish Research Council (2020-01164, grant holder: BJ), and Governmental Funding of Clinical Research within the National Health Service (grant holder: BJ). This work is part of the Danish nation-wide research program Childhood Oncology Network Targeting Research, Organization and Life expectancy (CONTROL) and supported by the Danish Cancer Society (R-257-A14720, grant holder: KS) and the Danish Childhood Cancer Foundation (2019-5934 and 2020-5769, grant holder: KS).

## Supplementary Material


