# Supplementary material for: Genetic Subtypes and Outcome of Patients Aged 1 to 45 Years Old With Acute Lymphoblastic Leukemia in the NOPHO ALL2008 Trial
Source: Hemasphere. 2023 May 4;7(5):e883. doi: 10.1097/HS9.0000000000000883 (PMC10162784; doi:10.1097/HS9.0000000000000883)
Supplement: Supplementary file 1 [file hs9-7-e883-s001.docx]

**Supplementary Information**

**The Nordic Society of Paediatric Haematology and Oncology (NOPHO) ALL2008 tria**l

This trial included patients aged between 1 and 45 years old with *BCR*::*ABL1*-negative B-cell precursor (BCP) or T-cell ALL in the Nordic countries, Estonia, and Lithuania [1-4]. The pediatric/adolescent (1-17 years) cohort was truly consecutive and population-based, whereas the inclusion of adults (>18 years) gradually increased during the trial; they were included from 2008 (Denmark), 2009 (Sweden), 2010 (Norway and Lithuania), 2011 (Estonia), and 2015 (Finland). The trial comprised a total of 1771 patients diagnosed between July 2008 and February 2016 (Supplementary Table 1). Follow-up data were extracted on September 14, 2021. The median observation time from diagnosis to last follow-up was 89.6 months (range, 0.7–157.2 months) for patients in continuous complete remission 1 (CR1; *n* = 1456) and 89.5 months (range, 0.7–157.2 months) for all patients alive at last follow-up (*n* = 1565). The median observation time from date of event to last follow up for those in remission after relapse or second malignant neoplasm (SMN) (n = 109) was 41.6 months (0–123.2 months).

**Genetic techniques used in the NOPHO ALL2008 trial**

At the start of the trial, conventional chromosome analysis (G-banding), DNA index measurements by flow cytometry, fluorescence in situ hybridization (FISH) with commercial probes, reverse-transcription polymerase chain reaction (RT-PCR) using validated primers, and Southern blot (for *KMT2A* rearrangements; *KMT2A*-r) were the accepted methods for ascertaining the presence of specific genetic subtypes. Southern blot analysis was only available in one diagnostic laboratory that also received samples from other NOPHO centers when their FISH results were difficult to interpret, *e.g.*, *KMT2A*-r-positive cases with concurrent 3’ deletions [5]. Single nucleotide polymorphism array (SNP-A) and array comparative genomic hybridization (aCGH) analyses were subsequently approved for detecting BCP ALL cases characterized by genomic imbalances, *i.e.*, intrachromosomal amplification of chromosome 21 (iAMP21), high hyperdiploidy (HeH), near-haploidy (NH), and low hypodiploidy (HoL). The genetic analyses were performed in 18 diagnostic centers and the findings in all cases were centrally reviewed annually by representatives from all centers.

**Definitions of genetic subtypes in the NOPHO ALL2008 trial**

***General rule*:** *BCR*::*ABL1*, *ETV6*::*RUNX1*, *TCF3*::*PBX1*, *KMT2A*-r, iAMP21, NH, and HoL were deemed mutually exclusive. Thus, if one of these was identified, the others were considered absent by default. HeH and dic(9;20)(p13;q11) were the exceptions because HeH occasionally (~3%) co-exists with *BCR*::*ABL1*, *ETV6*::*RUNX1*, *TCF3*::*PBX1*, or *KMT2A*-r and dic(9;20) may occur together with, for example, *BCR*::*ABL1* [6-8]. Cases with HeH or dic(9;20) with *BCR*::*ABL1*, *ETV6*::*RUNX1*, *TCF3*::*PBX1*, or *KMT2A*-r were grouped according to the latter, with the exception of dic(9;20)-positive cases with the *ETV6*::*RUNX1*-fusion. These were classified as dic(9;20) because *ETV6*::*RUNX1* was not a risk-stratifying abnormality in the ALL2008 protocol, whereas dic(9;20) was.

***Fusion genes*:** Conclusive positive or negative FISH or RT-PCR results for *BCR*::*ABL1*, *ETV6*::*RUNX1*, and *TCF3*::*PBX1* were sufficient to confirm or refute their presence. Chromosome banding analysis revealing t(1;19)(q23;p13)/der(19)t(1;19)(q23;p13) or t(9;22)(q34;q11) was insufficient because not all translocations with these breakpoints result in *TCF3*::*PBX1* or *BCR*::*ABL1*, respectively (https://mitelmandatabase.isb-cgc.org/). G-banding could not be used to detect the t(12;21)(p13;q22)/*ETV6*::*RUNX1* fusion because of the cryptic nature of this rearrangement.

***KMT2A-r***: FISH- or Southern blot-positivity or -negativity for *KMT2A*-r was enough to conclude if it was present or absent. It was not compulsory, although encouraged, to identify the *KMT2A* fusion partners. RT-PCR-positivity for a *KMT2A* fusion was also sufficient. RT-PCR-negativity did not exclude the presence of *KMT2A*-r due to the large number (~50) of 3’ partner genes known to fuse with *KMT2A* in ALL and cytogenetically identified 11q23 translocations were insufficient because there are several examples of 11q23 rearrangements not resulting in *KMT2A*-r (https://mitelmandatabase.isb-cgc.org/).

***iAMP21*:** Informative SNP-A or aCGH results with clear-cut data on the presence or absence of the typical 21q amplification pattern associated with iAMP21 [9], sufficed to confirm or refute the presence of this abnormality. Interphase FISH-negativity for extra signals of a *RUNX1* probe also excluded iAMP21. However, if three or more additional interphase signals for *RUNX1* were found, metaphase FISH analysis was compulsory to show that the supernumerary copies were present on a single abnormal chromosome 21 [10]. In the ALL2008 trial, one case, in which targeted analysis by FISH, SNP-A, and aCGH had not been performed, was nevertheless considered iAMP21-negative because the G-banding analysis had revealed an abnormal karyotype without any cytogenetic evidence for iAMP21, *i.e.*, an abnormal chromosome 21 or monosomy 21 together with a marker chromosome.

***dic(9;20)*:** This abnormality was judged to be present if it was detected by G-banding analysis, often using the presence of monosomy 20 and del(9p)/add(9p) as cytogenetic markers, or by a three-color FISH probe kit developed and validated for dic(9;20) [11, 12]. If SNP-A or aCGH revealed imbalances suggestive of dic(9;20), *e.g.*, loss of 9p13-pter and loss of 20q11-qter, both centromeres from chromosomes 9 and 20 had to be shown to co-localize by FISH or by cytogenetic scrutiny in order to conclude that this dicentric abnormality was present. The dic(9;20) was considered to be absent if the karyotype displayed no cytogenetic evidence/markers for this change or if FISH, SNP-A, or aCGH did not reveal any loss of the *CDKN2A*/*B* loci, which are always deleted as a consequence of dic(9;20) [13].

***HeH*:** High hyperdiploidy was defined as 51-67 chromosomes as identified by G-banding, SNP-A, or aCGH or as a DNA index between 1.12 and 1.35 [14, 15]. HeH was also considered present if interphase FISH analysis revealed at least five additional signals for chromosomes frequently gained in HeH, *i.e.*, X, 4, 6, 10, 14, 17, 18, and, always, 21 [6]. There should be no evidence of doubled-up NH or HoL clones and all targeted analyses for *BCR*::*ABL1*, *ETV6*::*RUNX1*, *TCF3*::*PBX1*, and *KMT2A*-r should be negative.

***NH*:** Near-haploidy was present if G-banding, SNP-A, or aCGH revealed a clone with 24-29 chromosomes or if the DNA index was below 0.60 (based on NH cases in previous NOPHO ALL protocols). Cases with doubled-up clones with 48-58 chromosomes were classified as NH [15, 16].

***HoL*:** Low hypodiploidy was defined as 30-39 chromosomes detected by G-banding, SNP-A, or aCGH or as a DNA index between 0.60-0.84 (based on HoL cases in previous NOPHO ALL protocols). Cases with doubled-up clones with 60-78 chromosomes were grouped as HoL [15, 16].

***B-other*** comprised BCP ALLs with confirmed absence of all the above-mentioned genetic subtypes. Cases without G-banding results could be classified as B-other only if all targeted analyses of all the above-mentioned genetic subtypes were negative.

***T-cell ALL:*** No T-cell-specific aberrations were screened for and, apart from *KMT2A*-r, the genetic subtypes defined above were only relevant in BCP ALL.

***Unknown:*** the definition of unknown, *i.e.*, genetically uninformative, differed between BCP and T-cell ALL. In BCP ALL, it consisted of karyotypic failures where one or more targeted analyses for the above-mentioned genetic subtypes had not been performed or had not provided clear-cut results. In T-cell ALL, it comprised karyotypic failures where analyses for *KMT2A*-r had not been done.

**Risk stratification in the NOPHO ALL 2008 trial**

At diagnosis, the patients were dichotomized to induction therapy with either dexamethasone (Dexa induction; Dex-I) or prednisolone (Pred induction; Pred-I); otherwise, the chemotherapy given was identical. The Dex-I group comprised BCP ALL patients with white blood cell (WBC) counts ≥100 x 10^9/^l and all T-cell ALL patients, whereas the Pred-I group consisted of BCP ALL patients with WBC counts <100 x 10^9/^l. After an amendment in 2009, patients with *ETV6*::*RUNX1*-positive ALL and WBC counts ≥100 x 10^9^/l were switched to Pred-I.

The second stratification, at the end of induction (EOI) on day 29, was primarily based on genetic findings and on the bone marrow measurable residual disease (MRD) status, resulting in three stratification groups, *viz.* high risk (HR), intermediate risk (IR), and standard risk (SR). The HR group comprised Dex-I patients with EOI MRD ≥0.1%, all cases with *KMT2A*-r, NH, or HoL, and Pred-I patients with EOI MRD >5%. The IR group consisted of Dex-I patients with EOI MRD <0.1%, Pred-I patients with EOI MRD ≥0.1% and <5%, and all cases with *TCF3*::*PBX1*, iAMP21, or dic(9;20)(p13;q11). The latter abnormality was risk-stratifying because dic(9;20)-positive cases had had a worse outcome compared with those with HeH or *ETV6*::*RUNX1* in the previous ALL1992 and ALL2000 trials [17, 18]. The SR group comprised Pred-I patients with EOI MRD <0.1%.

The third stratification transferred patients with SR and IR who had MRD >0.1% on day 79 to the HR group. In addition, the HR group was further subdivided, based on MRD findings EOI and after blocks A and B, into those treated with chemotherapy only (HR-chemo) and those intended for stem cell transplantation (HR-SCT).

The frequencies of the cytogenetic subgroups among the final risk groups were

SR: HeH (39%), *ETV6*::*RUNX1* (32%), and B-other (29%)

IR: HeH (28%), B-other (23%), *ETV6*::*RUNX1* (19%), *TCF3*::*PBX1* (9.0%), genetically uninformative cases (12%), dic(9;20) (5.2%), and iAMP21 (4.4%)

HR-chemo: *KMT2A*-r (44%), B-other (17%), HoL (14%), NH (8.1%), HeH (7.1%), genetically uninformative cases (5.1%), dic(9;20) (2.0%), and *ETV6*::*RUNX1* (2.0%)

HR-SCT: B-other (64%), HeH (14%), genetically uninformative cases (8.4%), *ETV6*::*RUNX1* (4.2%), iAMP21 (4.2%), *KMT2A*-r (3.2%), *TCF3*::*PBX1* (1.0%), and HoL (1.0%)

**Statistical analyses**

The IBM SPSS Statistics 27.0.1.0 software for Mac (IBM, New York, NY, USA) and the Stata Statistical Software Release 15 for Windows (College Station, StataCorp LLC, TX, USA) were used for statistical analyses. The significance limit for two-sided *P* values was set to <0.05. Differences in basic clinical data between the BCP and T-cell ALL patients and among the genetic subtypes and age groups were investigated by the chi-square or the Kruskal-Wallis one-way analysis of variance tests. The 5-year probabilities of disease-free survival (pDFS), event-free survival (pEFS), and overall survival (pOS) were ascertained using the Kaplan-Meier method, whereas the 5-year probability of cumulative incidence of relapse (pCIR), adjusted for the competing events death in CR1 (DCR1) and SMN, was determined by the command stcomlist in STATA 15 (https://ideas.repec.org/c/boc/bocode/s458292.html). The pCIR and pDFS were based on patients who achieved CR1 and were calculated from the date of CR1 to the date of first event (or last follow-up), with the event being relapse for pCIR and relapse, DCR1, or SMN for pDFS. The pEFS and pOS were calculated from the date of diagnosis to the first event (or last follow-up), *i.e.*, induction death, resistant disease, relapse, DCR1, and SMN for pEFS and death of any cause for pOS. Comparisons of pCIR, pDFS, pEFS, and pOS between the BCP and T-cell patients and among the genetic, age, and risk stratification groups were performed with the log-rank or weighted log-rank test.

In the multivariable analyses of pDFS and pOS, Cox regression models were used to analyze BCP ALL patients in the final stratification groups SR and IR. The following categorical variables were analyzed: MRD levels at EOI, the final treatment administered (SR, IR, HR (chemo and HSCT), and modified treatment), genetic subtypes, WBC counts (<50 and ≥50 x 10^9^/l; adhering to the NCI criteria), age groups (1-9, 10-17 and 18-45 years), central nervous system involvement, and two multiplicative interaction variables (one for the interaction between MRD day 29 and final treatment and one for the genetic subtype and final treatment). For various reasons (local clinician decisions, protocol violations, *et cetera*) 5.3% (68/1281) of the BCP ALL patients finally stratified to SR and IR, based on intention-to-treat, were in the end treated differently (Supplementary Table 15). Twenty patients had no final risk group: 18 with induction death, one lost to follow-up, one leaving protocol therapy before EOI, and two receiving such a modified therapy that they were considered outliers.

**References**

1. Toft N, Birgens H, Abrahamsson J, Bernell P, Griškevičius L, Hallböök H, et al. Risk group assignment differs for children and adults 1-45 yr with acute lymphoblastic leukemia treated by the NOPHO ALL-2008 protocol. Eur J Haematol. 2013;90:404-12.
2. Rank CU, Toft N, Tuckuviene R, Grell K, Nielsen OJ, Frandsen TL, et al. Thromboembolism in acute lymphoblastic leukemia: results of NOPHO ALL2008 protocol treatment in patients aged 1 to 45 years. Blood. 2018;131:2475-84.
3. Toft N, Birgens H, Abrahamsson J, Griškevičius L, Hallböök H, Heyman M, et al. Results of NOPHO ALL2008 treatment for patients aged 1-45 years with acute lymphoblastic leukemia. Leukemia. 2018;32:606-15.
4. Quist-Paulsen P, Toft N, Heyman M, Abrahamsson J, Griškevičius L, Hallböök H, et al. T-cell acute lymphoblastic leukemia in patients 1-45 years treated with the pediatric NOPHO ALL2008 protocol. Leukemia. 2020;34:347-57.
5. Barber KE, Ford AM, Harris RL, Harrison CJ, Moorman AV. MLL translocations with concurrent 3' deletions: interpretation of FISH results. Genes Chromosomes Cancer. 2004;41:266-71.
6. Paulsson K, Forestier E, Andersen MK, Autio K, Barbany G, Borgström G, et al. High modal number and triple trisomies are highly correlated favorable factors in childhood B-cell precursor high hyperdiploid acute lymphoblastic leukemia treated according to the NOPHO ALL 1992/2000 protocols. Haematologica. 2013;98:1424-32.
7. Rieder H, Schnittger S, Bodenstein H, Schwonzen M, Wörmann B, Berkovic D, et al. dic(9;20): a new recurrent chromosome abnormality in adult acute lymphoblastic leukemia. Genes Chromosomes Cancer. 1995;13:54-61.
8. Song X, Gong S, Yang J, Wang J. Clinical and molecular cytogenetic characteristics of dic(9;20) in adult acute lymphoblastic leukemia: a case report of three patients. Ann Hematol. 2007;86:347-51.
9. Strefford JC, van Delft FW, Robinson HM, Worley H, Yiannikouris O, Selzer R, et al. Complex genomic alterations and gene expression in acute lymphoblastic leukemia with intrachromosomal amplification of chromosome 21. Proc Natl Acad Sci U S A. 2006;103:8167-72.
10. Harrison CJ. Blood Spotlight on iAMP21 acute lymphoblastic leukemia (ALL), a high-risk pediatric disease. Blood. 2015;125:1383-6.
11. Clark R, Byatt SA, Bennett CF, Brama M, Martineau M, Moorman AV, et al. Monosomy 20 as a pointer to dicentric (9;20) in acute lymphoblastic leukemia. Leukemia. 2000;14:241-6.
12. Zachariadis V, Schoumans J, Öfverholm I, Barbany G, Halvardsson E, Forestier E, et al. Detecting dic(9;20)(p13.2;p11.2)-positive B-cell precursor acute lymphoblastic leukemia in a clinical setting using fluorescence in situ hybridization. Leukemia. 2014;28:196-8.
13. Zachariadis V, Schoumans J, Barbany G, Heyman M, Forestier E, Johansson B, et al. Homozygous deletions of CDKN2A are present in all dic(9;20)(p13·2;q11·2)-positive B-cell precursor acute lymphoblastic leukaemias and may be important for leukaemic transformation. Br J Haematol. 2012;159:488-91.
14. Forestier E, Holmgren G, Roos G. Flow cytometric DNA index and karyotype in childhood lymphoblastic leukemia. Anal Cell Pathol. 1998;17:145-56.
15. Paulsson K, Johansson B. High hyperdiploid childhood acute lymphoblastic leukemia. Genes Chromosomes Cancer. 2009;48:637-60.
16. Harrison CJ, Moorman AV, Broadfield ZJ, Cheung KL, Harris RL, Reza Jalali G, et al. Three distinct subgroups of hypodiploidy in acute lymphoblastic leukaemia. Br J Haematol. 2004;125:552-9.
17. Forestier E, Gauffin F, Andersen MK, Autio K, Borgström G, Golovleva I, et al. Clinical and cytogenetic features of pediatric dic(9;20)(p13.2;q11.2)-positive B-cell precursor acute lymphoblastic leukemias: a Nordic series of 24 cases and review of the literature. Genes Chromosomes Cancer. 2008;47:149-58.
18. Zachariadis V, Gauffin F, Kuchinskaya E, Heyman M, Schoumans J, Blennow E, et al. The frequency and prognostic impact of dic(9;20)(p13.2;q11.2) in childhood B-cell precursor acute lymphoblastic leukemia: results from the NOPHO ALL-2000 trial. Leukemia. 2011;25:622-8.

**Supplementary Table 1** The basic demographic, genetic, and clinical features of the 1771 BCP and T-cell ALL patients included in the NOPHO ALL2008 trial

| Variable | BCP ALL | T-cell ALL | *P* |
| --- | --- | --- | --- |
|  | *n* = 1493 (84%) | *n* = 278 (16%) |  |
| Median age in years (range) | 4.9 (1.0-45) | 12 (1.2-44) | <0.001^a^ |
| Age groups (years) |  |  |  |
| 1-9 | 1071 (72%) | 116 (42%) | <0.001^a^ |
| 10-17 | 227 (15%) | 79 (28%) |  |
| 18-45 | 195 (13%) | 83 (30%) |  |
| Sex |  |  |  |
| Female | 707 (47%) | 81 (29%) | <0.001^b^ |
| Male | 786 (53%) | 197 (71%) |  |
| Median WBC count x 10^9/^l (range) | 9.0 (0.0-1161) | 78 (1.0-983) | <0.001^a^ |
| <100 | 1378 (92%) | 157 (56%) | <0.001^a^ |
| ≥100 | 115 (7.7%) | 121 (44%) |  |
| CNS status |  |  |  |
| CNS1 | 1332 (89%) | 212 (76%) | <0.001^a^ |
| CNS2 | 115 (7.7%) | 31 (11%) |  |
| CNS3 | 42 (2.8%) | 30 (11%) |  |
| Missing | 4 (0.3%) | 5 (1.8%) |  |
| Genetic results^c^ |  |  |  |
| Abnormal | 1294 (87%) | 198 (71%) | 0.092^a^ |
| Normal | 123 (8.2%) | 50 (18%) |  |
| Unknown | 76 (5.1%) | 30 (11%) |  |
| Genetic subtypes |  |  |  |
| HeH | 471 (32%) | 0 | −^d^ |
| *ETV6*::*RUNX1* | 346 (23%) | 0 |  |
| *KMT2A*-r | 51 (3.4%) | 8 (2.9%) |  |
| *TCF3*::*PBX1* | 49 (3.3%) | 0 |  |
| dic(9;20)(p13;q11) | 29 (1.9%) | 0 |  |
| iAMP21 | 27 (1.8%) | 0 |  |
| HoL | 15 (1.0%) | 0 |  |
| NH | 8 (0.5%) | 0 |  |
| B-other | 421 (28%) | −^e^ |  |
| Unknown | 76 (5.1%) | 30 (11%) |  |
| Final stratification group^f^ |  |  |  |
| SR | 761 (52%) | 0 (0%) | <0.001^a^ |
| IR | 520 (35%) | 140 (51%) |  |
| HR-chemo | 99 (6.6%) | 107 (39%) |  |
| HR-SCT | 95 (6.4%) | 27 (9.7%) |  |
| Deaths | 136 (9.1%) | 70 (25%) | <0.001^b^ |
| Events | 241 (16%) | 74 (27%) | <0.001^b^ |
| Induction death | 14 (0.9%) | 4 (1.4%) |  |
| Resistant disease | 0 | 0 |  |
| DCR1 | 36 (2.4%) | 27 (9.7%) |  |
| Relapse | 178 (12%) | 42 (15%) |  |
| BM | 108 (61%) | 23 (55%) |  |
| CNS | 26 (15%) | 7 (17%) |  |
| Testicular | 3 (1.7%) | 1 (2.4%) |  |
| Other site | 7 (3.9%) | 2 (4.8%) |  |
| BM + CNS | 20 (11%) | 7 (17%) |  |
| BM + Testicular | 4 (2.2%) | 0 |  |
| BM + other site | 6 (3.4%) | 2 (4.8%) |  |
| BM, CNS + Testicular | 4 (2.2%) | 0 |  |
| SMN | 13 (0.9%) | 1 (0.4%) |  |
| pCIR at 5 years ± SE (no. of relapses) | 0.11 ± 0.01 (158) | 0.15 ± 0.02 (40) | 0.093^g^ |
| pDFS at 5 years ± SE (no. of events) | 0.85 ± 0.01 (206) | 0.75 ± 0.03 (68) | <0.001^h^ |
| pEFS at 5 years ± SE (no. of events) | 0.85 ± 0.01 (219) | 0.74 ± 0.03 (71) | <0.001^h^ |
| pOS at 5 years ± SE (no. of deaths) | 0.92 ± 0.01 (120) | 0.75 ± 0.03 (68) | <0.001^h^ |

ALL, acute lymphoblastic leukemia; BCP, B-cell precursor; BM, bone marrow; CNS, central nervous

system; CNS1, no blasts on cytospin and no clinical signs of CNS leukemia; CNS2, >0 and <5 cells/µl cerebrospinal fluid that on cytospin were regarded to represent leukemic blasts but no other signs of CNS leukemia; CNS3, ≥5 cells/µl cerebrospinal fluid that on cytospin were regarded to represent leukemic blasts and/or signs of CNS leukemia; DCR1, death in first complete remission; HeH, high hyperdiploidy (51-67 chromosomes); HoL, low hypodiploidy (30-39 chromosomes); HR-chemo, high-risk patients stratified to treatment with chemotherapy only; HR-SCT, high-risk patients stratified to stem cell transplantation; iAMP21, intrachromosomal amplification of chromosome 21; IR, intermediate risk; *KMT2A*-r, *KMT2A* rearrangement; *n*, number of patients; NH, near-haploidy (24-29 chromosomes); pCIR, probability of cumulative incidence of relapse; pDFS, probability of disease-free survival; pEFS, probability of event-free survival; pOS, probability of overall survival; SE, standard error; SMN, secondary malignant neoplasm; SR, standard risk; WBC, white blood cell.

^a^Kruskal-Wallis one-way analysis of variance test.

^b^Chi-square test.

^c^Based on all genetic methods used (conventional chromosome banding, DNA-index, fluorescence in situ hybridization, reverse-transcription polymerase chain reaction, Southern blot, single nucleotide polymorphism array, and/or array comparative genomic hybridization analyses).

^d^Not applicable because the genetic subtypes screened for, except *KMT2A*-r, were only relevant in BCP ALL.

^e^Not applicable because T-cell ALL cannot, by definition, be grouped as B-other.

^f^Of the 1493 BCP ALL patients, 18 had no final risk group (14 with induction death, one lost to follow-up, one leaving protocol therapy before day 29, and two receiving such a modified therapy that they were considered outliers) and of the 278 T-cell ALL patients, four were not finally stratified because of induction death. Thus, the final risk groups of the BCP and T-cell ALLs comprised 1475 and 274 patients, respectively.

^g^Weighted log-rank test.

^h^Log-rank test.

**Supplementary Table 2** The 278 T-cell ALL patients in relation to genetic results

| Variable | Genetic results^a^ | | | | *P* |
| --- | --- | --- | --- | --- | --- |
|  | | Normal | Abnormal | Unknown |  |
|  | | *n* = 50 (18%) | *n* = 198 (71%) | *n* = 30 (11%) |  |
| Median age (range) | | 10.8 (2.0-43) | 12.5 (1.3-44) | 14.1 (1.2-41) | 0.680^b^ |
| Age groups | |  |  |  |  |
| 1-9 | | 21 (42%) | 84 (42%) | 11 (37%) | 0.467^b^ |
| 10-17 | | 14 (28%) | 59 (30%) | 6 (20%) |  |
| 18-45 | | 15 (30%) | 55 (28%) | 13 (43%) |  |
| Sex | |  |  |  |  |
| Female | | 15 (30%) | 56 (28%) | 10 (33%) | 0.843^c^ |
| Male | | 35 (70%) | 142 (72%) | 20 (67%) |  |
| Median WBC count x 10^9/^l (range) | | 68 (1.0-757) | 91 (1.0-983) | 51 (1.0-825) | 0.464^b^ |
| <100 | | 34 (68%) | 103 (52%) | 20 (67%) | 0.062^b^ |
| ≥100 | | 16 (32%) | 95 (48%) | 10 (33%) |  |
| CNS status | |  |  |  |  |
| CNS1 | | 35 (70%) | 150 (76%) | 27 (90%) | 0.151^b^ |
| CNS2 | | 9 (18%) | 21 (11%) | 1 (3.3%) |  |
| CNS3 | | 5 (10%) | 23 (12%) | 2 (6.7%) |  |
| Missing | | 1 (2.0%) | 4 (2.0%) | 0 |  |
| Stratification on day 29^d^ | | *n* = 49 | *n* = 195 | *n* = 30 | 0.225^b^ |
| SR | | −^e^ | −^e^ | −^e^ |  |
| IR | | 31 (63%) | 92 (47%) | 17 (57%) |  |
| HR-chemo | | 14 (29%) | 91 (47%) | 10 (33%) |  |
| HR-SCT | | 4 (8.2%) | 12 (6.2%) | 3 (10%) |  |
| Final stratification^d^ | | *n* = 49 | *n* = 195 | *n* = 30 | 0.174^b^ |
| SR | | −^e^ | −^e^ | −^e^ |  |
| IR | | 31 (63%) | 92 (47%) | 17 (57%) |  |
| HR-chemo | | 12 (24%) | 83 (43%) | 12 (40%) |  |
| HR-SCT | | 6 (12%) | 20 (10%) | 1 (3.3%) |  |
| Deaths | | 12 (24%) | 50 (25%) | 8 (27%) | 0.964^b^ |
| Events | | 13 (26%) | 53 (27%) | 8 (27%) | 0.994^b^ |
| Induction death | | 1 (2.0%) | 3 (1.5%) | 0 |  |
| Resistant disease | | 0 | 0 | 0 |  |
| DCR1 | | 7 (14%) | 19 (9.6%) | 1 (3.3%) |  |
| Relapse | | 5 (10%) | 30 (15%) | 7 (23%) |  |
| BM | | 4 (80%) | 15 (50%) | 4 (57%) |  |
| CNS | | 0 | 5 (17%) | 2 (29%) |  |
| Testicular | | 0 | 1 (3.3%) | 0 |  |
| Other site | | 0 | 2 (6.7%) | 0 |  |
| BM + CNS | | 0 | 6 (20%) | 1 (14%) |  |
| BM + Testicular | | 0 | 0 | 0 |  |
| BM + other site | | 1 (20%) | 1 (3.3%) | 0 |  |
| BM, CNS + Testicular | | 0 | 0 | 0 |  |
| SMN | | 0 | 1 (0.5%) | 0 |  |
| pCIR at 5 years ± SE (no. of relapses) | | 0.08 ± 0.04 (4) | 0.15 ± 0.03 (29) | 0.23 ± 0.08 (7) | 0.276^f^ |
| pDFS at 5 years ± SE (no. of events) | | 0.78 ± 0.06 (11) | 0.75 ± 0.03 (49) | 0.73 ± 0.08 (8) | 0.983^g^ |
| pEFS at 5 years ± SE (no. of events) | | 0.76 ± 0.06 (12) | 0.73 ± 0.03 (52) | 0.77 ± 0.08 (7) | 0.980^g^ |
| pOS at 5 years ± SE (no. of deaths) | | 0.76 ± 0.06 (12) | 0.75 ± 0.03 (49) | 0.77 ± 0.08 (7) | 0.994^g^ |

ALL, acute lymphoblastic leukemia; BM, bone marrow; CNS, central nervous system; CNS1, no

blasts on cytospin and no clinical signs of CNS leukemia; CNS2, >0 and <5 cells/µl cerebrospinal fluid that on cytospin were regarded to represent leukemic blasts but no other signs of CNS leukemia; CNS3, ≥5 cells/µl cerebrospinal fluid that on cytospin were regarded to represent leukemic blasts and/or signs of CNS leukemia; DCR1, death in first complete remission; HR-chemo, high-risk patients stratified to treatment with chemotherapy only; HR-SCT, high-risk patients stratified to stem cell transplantation; IR, intermediate risk; *n*, number of patients; pCIR, probability of cumulative incidence of relapse; pDFS, probability of disease-free survival; pEFS, probability of event-free survival; pOS, probability of overall survival; SE, standard error; SMN, secondary malignant neoplasm; SR, standard risk; WBC, white blood cell.

^a^Based on all genetic methods used (conventional chromosome banding, DNA-index, fluorescence in situ hybridization, reverse-transcription polymerase chain reaction, Southern blot, single nucleotide polymorphism array, and/or array comparative genomic hybridization analyses).

^b^Kruskal-Wallis one-way analysis of variance test.

^c^Chi-square test.

^d^Of the 278 T-cell ALL patients, four were not stratified after diagnosis because of induction death.

^e^Not applicable because T-cell patients could not be stratified as SR.

^f^Weighted log-rank test.

^g^Log-rank test.

**Supplementary Table 3** Outcome of children and adults with T-cell ALL in the NOPHO ALL2008 trial and in the literature

| Trial | MRD- | Age | 5-year^a^ survival (SE or CI) | | | | Reference |
| --- | --- | --- | --- | --- | --- | --- | --- |
|  | based | group | pCIR | pDFS | pEFS | pOS |  |
|  |  | (years) |  |  |  |  |  |
| NOPHO ALL2008 | Yes | 1-45 | 0.15 (0.02) | 0.75 (0.03) | 0.74 (0.03) | 0.75 (0.03) | Present |
| NOPHO ALL2008 | Yes | 1-9 | 0.14 (0.03) | 0.81 (0.04) | 0.80 (0.04) | 0.82 (0.04) | Present |
| NOPHO ALL2008 | Yes | 10-17 | 0.08 (0.03) | 0.78 (0.05) | 0.76 (0.05) | 0.75 (0.05) | Present |
| NOPHO ALL2008 | Yes | 18-45 | 0.22 (0.05) | 0.64 (0.05) | 0.65 (0.05) | 0.66 (0.05) | Present |
| NOPHO ALL2000 | No | 1-15 | NR | NR | 0.64 (0.05) | 0.72 (0.01) | 1 |
| AIEOP-BFM ALL | Yes | 1-17 | NR | NR | 0.763 (0.02) | 0.812 (0.018) | 2 |
| 2000/R2006 |  |  |  |  |  |  |  |
| CoALL 07-03 | Yes | 1-18 | NR | NR | 0.808 (0.038) | 0.862 (0.033) | 3 |
| COG AALL0434 | Yes | 1-31 | NR | 0.915 (0.881-0.948) | 0.838 (0.812-0.864) | 0.937 (0.908-0.966) | 4 |
| DCOG ALL10 | Yes | 1-18 | 0.072 (0.021) | NR | 0.800 (0.037) | 0.952 (0.017) | 5 |
| EORTC-CLG 58881 | No | 0-17 | NR | 0.673 (0.028)^b^ | 0.651 (0.026)^b^ | 0.719 (0.026)^b^ | 6 |
| EORTC-CLG 58951 | Yes | 0-17 | NR | 0.758 (0.026)^b^ | 0.740 (0.026)^b^ | 0.782 (0.025)^b^ | 6 |
| GRAALL 2003 | Yes | 15-60 | 0.31 (0.25-0.38)^c^ | 0.63 (0.51-0.73%)^c^ | 0.57 (0.50-0.63)^c^ | 0.61 (0.54-0.68)^c^ | 7 |
| MRC UKALLXII/ | No | 15-59 | 0.42 (0.36-0.47) | NR | NR | 0.48 (0.42-0.53) | 8 |
| ECOG 2993 |  |  |  |  |  |  |  |
| PETHEMA HR- | Yes | 15-60 | 0.41 (0.31‐0.51)^d^ | NR | 0.38 (0.29‐0.47)^d^ | 0.44 (0.34‐0.54)^d^ | 9 |
| ALL03 |  |  |  |  |  |  |  |
| PETHEMA HR- | Yes | 15-60 | 0.49 (0.33‐0.64)^d^ | NR | 0.46 (0.32‐0.60)^d^ | 0.65 (0.51‐0.79)^d^ | 9 |
| ALL11 |  |  |  |  |  |  |  |
| St Jude Total | Yes | <18 | NR | NR | 0.810 (0.718-0.902) | 0.870 (0.790-0.950) | 10 |
| Therapy Study 16 |  |  |  |  |  |  |  |
| TCCSG L04-16 | No | 1-17 | NR | NR | 0.620 (0.046) | 0.719 (0.043) | 11 |
| TPOG‐ALL‐2013 | Yes | <18 | 0.283 (0.127-0.462) | NR | 0.549 (0.353-0.708) | 0.634 (0.398-0.799) | 12 |
| UKALL2003 | Yes | 1-24 | NR | NR | 0.792 (0.748-0.829)^e^ | 0.849 (0.809-0.881)^e^ | 13 |

ALL, acute lymphoblastic leukemia; CI, confidence interval; MRD, measurable residual disease; NR, not reported; pCIR, probability of cumulative incidence

of relapse; pDFS, probability of disease-free survival; pEFS, probability of event-free survival; pOS, probability of overall survival; SE, standard error.

^a^Unless otherwise specified.

^b^At 8 years.

^c^At 3.5 years.

^d^At 2 years.

^e^At 10 years.

**References**

1. Schmiegelow K, Forestier E, Hellebostad M, Heyman M, Kristinsson J, Söderhäll S, et al. Long-term results of NOPHO ALL-92 and ALL-2000 studies of childhood acute lymphoblastic leukemia. Leukemia. 2010;24:345-54.

2. Schrappe M, Valsecchi MG, Bartram CR, Schrauder A, Panzer-Grümayer R, Möricke A, et al. Late MRD response determines relapse risk overall and in subsets of childhood T-cell ALL: results of the AIEOP-BFM-ALL 2000 study. Blood. 2011;118:2077-84.

3. Schramm F, zur Stadt U, Zimmermann M, Jorch N, Pekrun A, Borkhardt A, et al. Results of CoALL 07-03 study childhood ALL based on combined risk assessment by in vivo and in vitro pharmacosensitivity. Blood Adv. 2019;3:3688-99.

4. Winter SS, Dunsmore KP, Devidas M, Wood BL, Esiashvili N, Chen Z, et al. Improved survival for children and young adults with T-lineage acute lymphoblastic leukemia: results from the Children’s Oncology Group AALL0434 methotrexate randomization. J Clin Oncol. 2018;36:2926-34.

5. Pieters R, de Groot-Kruseman H, Van der Velden V, Fiocco M, van den Berg H, de Bont E, et al. Successful therapy reduction and intensification for childhood acute lymphoblastic leukemia based on minimal residual disease monitoring: study ALL10 from the Dutch Childhood Oncology Group. J Clin Oncol. 2016;34:2591-601.

6. Hofmans M, Suciu S, Ferster A, Van Vlierberghe P, Mazingue F, Sirvent N, et al. Results of successive EORTC-CLG 58 881 and 58 951 trials in paediatric T-cell acute lymphoblastic leukaemia (ALL). Br J Haematol. 2019;186:741-53.

7. Huguet F, Leguay T, Raffoux E, Thomas X, Beldjord K, Delabesse E, et al. Pediatric-inspired therapy in adults with Philadelphia chromosome-negative acute lymphoblastic leukemia: the GRAALL-2003 study. J Clin Oncol. 2009;27:911-8.

8. Marks DI, Paietta EM, Moorman AV, Richards SM, Buck G, DeWald G, et al. T-cell acute lymphoblastic leukemia in adults: clinical features, immunophenotype, cytogenetics, and outcome from the large randomized prospective trial (UKALL XII/ECOG 2993). Blood. 2009;114:5136-45.

9. Barba P, Morgades M, Montesinos P, Gil C, Fox M-L, Ciudad J, et al. Increased survival due to lower toxicity for high-risk T-cell acute lymphoblastic leukemia patients in two consecutive pediatric-inspired PETHEMA trials. Eur J Haematol. 2019;102:79-86.

10. Jeha S, Pei D, Choi J, Cheng C, Sandlund JT, Coustan-Smith E, et al. Improved CNS control of childhood acute lymphoblastic leukemia without cranial irradiation: St Jude Total Therapy Study 16. J Clin Oncol. 2019;37:3377-91.

11. Takahashi H, Kajiwara R, Kato M, Hasegawa D, Tomizawa D, Noguchi Y, et al. Treatment outcome of children with acute lymphoblastic leukemia: the Tokyo Children’s Cancer Study Group (TCCSG) Study L04-16. Int J Hematol. 2018;108:98-108.

12. Yu CH, Jou ST, Su YH, Coustan-Smith E, Wu G, Cheng CN, et al. Clinical impact of minimal residual disease and genetic subtypes on the prognosis of childhood acute lymphoblastic leukemia. Cancer (in press).

13. Moorman AV, Antony G, Wade R, Butler ER, Enshaei A, Harrison CJ, et al. Time to cure for childhood and young adult acute lymphoblastic leukemia is independent of early risk factors: Long-term follow-up of the UKALL2003 trial. J Clin Oncol. 2022;40:4228-39.

**Supplementary Table 4** The eight T-cell ALL cases with *KMT2A*-r

| Age | Sex | WBC | CNS | Partner | Final | Event | Survival |
| --- | --- | --- | --- | --- | --- | --- | --- |
| (years) |  | count | status | gene/ | stratification |  | in months |
|  |  | (x 10^9^/l) |  | locus |  |  |  |
| 2.9 | M | 183 | CNS1 | *MLLT1* | HR-SCT | None | 141+ in CR1 |
| 3.6 | M | 258 | CNS1 | 2p21 | HR-SCT | None | 42+ in CR1 |
| 10.9 | F | 11 | CNS2 | Unknown | HR-chemo | None | 100+ in CR1 |
| 14.0 | M | 28 | CNS1 | 19p13 | HR-chemo | None | 83+ in CR1 |
| 15.2 | M | 2 | CNS1 | *AFF1* | HR-chemo | DCR1 | 16 |
| 17.4 | F | 15 | CNS1 | Unknown | HR-chemo | None | 62+ in CR1 |
| 21.8 | F | 5 | CNS1 | *CBL* | HR-chemo | None | 41+ in CR1 |
| 36.9 | M | 305 | CNS1 | 19p13 | HR-chemo | None | 59+ in CR1 |

ALL, acute lymphoblastic leukemia; CNS, central nervous system; CNS1, no blasts on cytospin

and no clinical signs of CNS leukemia; CNS2, >0 and <5 cells/µl cerebrospinal fluid that on cytospin were regarded to represent leukemic blasts but no other signs of CNS leukemia; CR1, first complete remission; DCR1, death in CR1; F, female; HR-chemo, high-risk patients stratified to treatment with chemotherapy only; HR-SCT, high-risk patients stratified to stem cell transplantation; *KMT2A*-r, *KMT2A* rearrangement; M, male; WBC, white blood cell; + alive at last follow-up (September 14, 2021).

**Supplementary Table 5** 5-year survival of the genetic subtypes in relation to final risk stratification groups of the BCP ALL patients

| Genetic subtype | SR | IR | HR-chemo | HR-SCT |
| --- | --- | --- | --- | --- |
|  | *n* = 761 | *n* = 520 | *n* = 99 | *n* = 95 |
| HeH (*n* = 465) | *n* = 301 | *n* = 144 | *n* = 7 | *n* = 13 |
| pCIR (no. of relapses) ± SE | 0.03 ± 0.01 (9) | 0.09 ± 0.02 (13) | 0.14 ± 0.13 (1) | 0.09 ± 0.09 (1) |
| pDFS (no. of events) ± SE | 0.94 ± 0.01 (17) | 0.90 ± 0.03 (14) | 0.86 ± 0.13 (1) | 0.83 ± 0.11 (2) |
| pEFS (no. of events) ± SE | 0.94 ± 0.01 (17) | 0.88 ± 0.03 (14) | 0.86 ± 0.13 (1) | 0.83 ± 0.11 (2) |
| pOS (no. of deaths) ± SE | 0.99 ± 0.01 (4) | 0.96 ± 0.02 (5) | 0.86 ± 0.13 (1) | 0.92 ± 0.07 (1) |
|  |  |  |  |  |
| *ETV6*::*RUNX1* (*n* = 345) | *n* = 240 | *n* = 99 | *n* = 2 | *n* = 4 |
| pCIR (no. of relapses) ± SE | 0.04 ± 0.01 (8) | 0.11 ± 0.03 (10) | 0 ± 0 (0) | 0.50 ± 0.25 (2) |
| pDFS (no. of events) ± SE | 0.95 ± 0.02 (12) | 0.85 ± 0.04 (14) | 1.0 ± 0 (0) | 0.50 ± 0.25 (2) |
| pEFS (no. of events) ± SE | 0.95 ± 0.02 (12) | 0.86 ± 0.04 (13) | 1.0 ± 0 (0) | 0.50 ± 0.25 (2) |
| pOS (no. of deaths) ± SE | 0.99 ± 0.01 (3) | 0.96 ± 0.02 (4) | 1.0 ± 0 (0) | 0.50 ± 0.25 (2) |
|  |  |  |  |  |
| *TCF3*::*PBX1* (*n* = 48) | *n* = 0 | *n* = 47 | *n* = 0 | *n* = 1 |
| pCIR (no. of relapses) ± SE | − | 0.02 ± 0.02 (1) | − | 0 ± 0 (0) |
| pDFS (no. of events) ± SE | − | 0.96 ± 0.03 (2) | − | 1.0 ± 0 (0) |
| pEFS (no. of events) ± SE | − | 0.96 ± 0.03 (2) | − | 1.0 ± 0 (0) |
| pOS (no. of deaths) ± SE | − | 0.98 ± 0.02 (1) | − | 1.0 ± 0 (0) |
|  |  |  |  |  |
| *KMT2A*-r (*n* = 47) | *n* = 0 | *n* = 0 | *n* = 44 | *n* = 3 |
| pCIR (no. of relapses) ± SE | − | − | 0.14 ± 0.05 (6) | 1.0 ± 0.27 (3) |
| pDFS (no. of events) ± SE | − | − | 0.75 ± 0.06 (11) | 0 ± 0 (3) |
| pEFS (no. of events) ± SE | − | − | 0.75 ± 0.06 (11) | 0 ± 0 (3) |
| pOS (no. of deaths) ± SE | − | − | 0.77 ± 0.06 (10) | 0.33 ± 0.27 (2) |
|  |  |  |  |  |
| dic(9;20) (*n* = 29) | *n* = 0 | *n* = 27 | *n* = 2 | *n* = 0 |
| pCIR (no. of relapses) ± SE | − | 0.07 ± 0.05 (2) | 1.0 ± 0 (2) | − |
| pDFS (no. of events) ± SE | − | 0.89 ± 0.06 (3) | 0 ± 0 (2) | − |
| pEFS (no. of events) ± SE | − | 0.89 ± 0.06 (3) | 0 ± 0 (2) | − |
| pOS (no. of deaths) ± SE | − | 0.96 ± 0.04 (1) | 0 ± 0 (2) | − |
|  |  |  |  |  |
| iAMP21 (*n* = 27) | *n* = 0 | *n* = 23 | *n* = 0 | *n* = 4 |
| pCIR (no. of relapses) ± SE | − | 0.36 ± 0.10 (8) | − | 0 ± 0 (0) |
| pDFS (no. of events) ± SE | − | 0.64 ± 0.10 (8) | − | 0.75 ± 0.22 (1) |
| pEFS (no. of events) ± SE | − | 0.64 ± 0.10 (8) | − | 0.75 ± 0.22 (1) |
| pOS (no. of deaths) ± SE | − | 0.95 ± 0.05 (1) | − | 0.75 ± 0.22 (1) |
|  |  |  |  |  |
| HoL (*n* = 15) | *n* = 0 | *n* = 0 | *n* = 14 | *n* = 1 |
| pCIR (no. of relapses) ± SE | − | − | 0.21 ± 0.11 (3) | 1.0 ± 0 (1) |
| pDFS (no. of events) ± SE | − | − | 0.64 ± 0.13 (5) | 0 ± 0 (1) |
| pEFS (no. of events) ± SE | − | − | 0.64 ± 0.13 (5) | 0 ± 0 (1) |
| pOS (no. of deaths) ± SE | − | − | 0.71 ± 0.12 (4) | 0 ± 0 (1) |
|  |  |  |  |  |
| NH (*n* = 8) | *n* = 0 | *n* = 0 | *n* = 8 | *n* = 0 |
| pCIR (no. of relapses) ± SE | − | − | 0.12 ± 0.12 (1) | − |
| pDFS (no. of events) ± SE | − | − | 0.50 ± 0.18 (4) | − |
| pEFS (no. of events) ± SE | − | − | 0.50 ± 0.18 (4) | − |
| pOS (no. of deaths) ± SE | − | − | 0.50 ± 0.18 (4) | − |
|  |  |  |  |  |
| B-other (*n* = 418) | *n* = 220 | *n* = 120 | *n* = 17 | *n* = 61 |
| pCIR (no. of relapses) ± SE | 0.14 ± 0.02 (28) | 0.22 ± 0.04 (25) | 0.41 ± 0.12 (7) | 0.23 ± 0.05 (14) |
| pDFS (no. of events) ± SE | 0.84 ± 0.03 (33) | 0.76 ± 0.04 (27) | 0.47 ± 0.12 (9) | 0.69 ± 0.06 (19) |
| pEFS (no. of events) ± SE | 0.84 ± 0.03 (33) | 0.76 ± 0.04 (27) | 0.47 ± 0.12 (9) | 0.69 ± 0.06 (19) |
| pOS (no. of deaths) ± SE | 0.92 ± 0.02 (16) | 0.86 ± 0.03 (16) | 0.53 ± 0.12 (8) | 0.79 ± 0.05 (13) |
|  |  |  |  |  |
| Unknown (*n* = 73) | *n* = 0 | *n* = 60 | *n* = 5 | *n* = 8 |
| pCIR (no. of relapses) ± SE | − | 0.14 ± 0.05 (8) | 0.40 ± 0.22 (2) | 0.25 ± 0.15 (2) |
| pDFS (no. of events) ± SE | − | 0.82 ± 0.05 (10) | 0.60 ± 0.22 (2) | 0.75 ± 0.15 (2) |
| pEFS (no. of events) ± SE | − | 0.82 ± 0.05 (10) | 0.53 ± 0.25 (2) | 0.75 ± 0.15 (2) |
| pOS (no. of deaths) ± SE | − | 0.93 ± 0.03 (4) | 1.0 ± 0 (0) | 0.75 ± 0.15 (2) |

BCP ALL, B-cell precursor acute lymphoblastic leukemia; HeH, high hyperdiploidy (51-67

chromosomes); HoL, low hypodiploidy (30-39 chromosomes); HR-chemo, high-risk patients stratified to treatment with chemotherapy only; HR-SCT, high-risk patients stratified to stem cell transplantation; iAMP21, intrachromosomal amplification of chromosome 21; IR, intermediate risk; *KMT2A*-r, *KMT2A* rearrangement; *n*, number of patients; NH, near-haploidy (24-29 chromosomes); pCIR, probability of cumulative incidence of relapse; pDFS, probability of disease-free survival; pEFS, probability of event-free survival; pOS, probability of overall survival; SE, standard error; SR, standard risk.

**Supplementary Table 6** Outcome of children and adults with high hyperdiploid BCP ALL in the NOPHO ALL2008 trial and in the literature

| Trial | MRD- | Age | 5-year^a^ survival (SE or CI) | | | | Reference |
| --- | --- | --- | --- | --- | --- | --- | --- |
|  | based | group | pCIR | pDFS | pEFS | pOS |  |
|  |  | (years) |  |  |  |  |  |
| NOPHO ALL2008 | Yes | 1-45 | 0.06 (0.01) | 0.92 (0.01) | 0.91 (0.02) | 0.97 (0.01) | Present |
| NOPHO ALL2008 | Yes | 1-9 | 0.05 (0.01) | 0.93 (0.01) | 0.92 (0.01) | 0.96 (0.01) | Present |
| NOPHO ALL2008 | Yes | 10-17 | 0.08 (0.04) | 0.92 (0.04) | 0.92 (0.04) | 0.98 (0.02) | Present |
| NOPHO ALL2008 | Yes | 18-45 | 0.12 (0.08) | 0.82 (0.10) | 0.82 (0.10) | 0.95 (0.07) | Present |
| NOPHO ALL2000 | No | 1-15 | NR | NR | 0.84 (0.02) | 0.90 (0.01) | 1 |
| NOPHO ALL1992/ | No | 1-15 | NR | NR | 0.82 (0.02) | 0.91 (0.01) | 2 |
| 2000 |  |  |  |  |  |  |  |
| ALL-BFM-A 2000 | Yes | <18 | NR | NR | 0.828 (0.034) | 0.900 (0.027) | 3 |
| DCOG ALL10 | Yes | 1-18 | 0.089 (0.022) | NR | 0.879 (0.025) | 0.933 (0.019) | 4 |
| DFCI 05-001 | Yes | 1-18 | NR | NR | 0.89 (0.84-0.93) | NR | 5 |
| EORTC-CLG | Yes | 0-17 | NR | NR | 0.890 (0.015)^b^ | 0.959 (0.0.9)^b^ | 6 |
| 58951 |  |  |  |  |  |  |  |
| GRAALL 2003/ | Partly | 15-59 | NR | NR | 0.63 (0.45-0.77) | 0.72 (0.54-0.84) | 7 |
| 2005 |  |  |  |  |  |  |  |
| MRC ALL97/99 | No | 1-18 | NR | NR | 0.84 (0.81-0.87) | 0.93 (0.91-0.95) | 8 |
| MRC UKALLXII/ | No | 15-65 | NR | NR | 0.50 (0.38-0.60) | 0.53 (0.41-0.64) | 9 |
| ECOG 2993 |  |  |  |  |  |  |  |
| St Jude Total | Yes | <18 | NR | NR | 0.947 (0.900-0.994) | 0.994 (0.976-1.00) | 10 |
| Therapy Study 16 |  |  |  |  |  |  |  |
| TCCSG L04-16 | No | 1-17 | NR | NR | 0.850 (0.027) | 0.954 (0.016) | 11 |
| TPOG-ALL-2013 | Yes | <18 | 0.081 (0.024-0.185) | NR | 0.895 (0.784-0.951) | 0.946 (0.793-0.987) | 12 |
| UKALL2003 | Yes | 1-24 | NR | NR | 0.900 (0.878-0.919)^c^ | 0.941 (0.922-0.955)^c^ | 13 |
| UKALL14 | Yes | 25-65 | NR | NR | 0.54 (0.25-0.76)^d^ | 0.54 (0.25-0.76)^d^ | 14 |

BCP ALL, B-cell precursor acute lymphoblastic leukemia; CI, confidence interval; MRD, measurable residual disease; NR, not reported; pCIR, probability of

cumulative incidence of relapse; pDFS, probability of disease-free survival; pEFS, probability of event-free survival; pOS, probability of overall survival; SE, standard error.

^a^Unless otherwise specified.

^b^At 6 years.

^c^At 10 years.

^d^At 3 years.

**References**

1. Schmiegelow K, Forestier E, Hellebostad M, Heyman M, Kristinsson J, Söderhäll S, et al. Long-term results of NOPHO ALL-92 and ALL-2000 studies of childhood acute lymphoblastic leukemia. Leukemia. 2010;24:345-54.

2. Paulsson K, Forestier E, Andersen MK, Autio K, Barbany G, Borgström G, et al. High modal number and triple trisomies are highly correlated favorable factors in childhood B-cell precursor high hyperdiploid acute lymphoblastic leukemia treated according to the NOPHO ALL 1992/2000 protocols. Haematologica. 2013;98:1424-32.

3. Reismüller B, Steiner M, Pichler H, Dworzak M, Urban C, Meister B, et al. High hyperdiploid acute lymphoblastic leukemia (ALL) - a 25-year population-based survey of the Austrian ALL-BFM (Berlin-Frankfurt-Münster) Study Group. Pediatr Blood Cancer. 2017;64:e26327.

4. Pieters R, de Groot-Kruseman H, Van der Velden V, Fiocco M, van den Berg H, de Bont E, et al. Successful therapy reduction and intensification for childhood acute lymphoblastic leukemia based on minimal residual disease monitoring: study ALL10 from the Dutch Childhood Oncology Group. J Clin Oncol. 2016;34:2591-601.

5. Vrooman LM, Blonquist TM, Harris MH, Stevenson KE, Place AE, Hunt SK, et al. Refining risk classification in childhood B acute lymphoblastic leukemia: results of DFCI ALL Consortium Protocol 05-001. Blood Adv. 2018;2:1449-58.

6. Dastugue N, Suciu S, Plat G, Speleman F, Cavé H, Girard S, et al. Hyperdiploidy with 58-66 chromosomes in childhood B-acute lymphoblastic leukemia is highly curable: 58951 CLG-EORTC results. Blood. 2013;121:2415-23.

7. Lafage-Pochitaloff M, Baranger L, Hunault M, Cuccuini W, Lefebvre C, Bidet A, et al. Impact of cytogenetic abnormalities in adults with Ph-negative B-cell precursor acute lymphoblastic leukemia. Blood. 2017;130:1832-44.

8. Moorman AV, Ensor HM, Richards SM, Chilton L, Schwab C, Kinsey SE, et al. Prognostic effect of chromosomal abnormalities in childhood B-cell precursor acute lymphoblastic leukaemia: results from the UK Medical Research Council ALL97/99 randomised trial. Lancet Oncol. 2010;11:429-38.

9. Moorman AV, Harrison CJ, Buck GAN, Richards SM, Secker-Walker LM, Martineau M, et al. Karyotype is an independent prognostic factor in adult acute lymphoblastic leukemia (ALL): analysis of cytogenetic data from patients treated on the Medical Research Council (MRC) UKALLXII/Eastern Cooperative Oncology Group (ECOG) 2993 trial. Blood. 2007;109:3189-97.

10. Jeha S, Pei D, Choi J, Cheng C, Sandlund JT, Coustan-Smith E, et al. Improved CNS control of childhood acute lymphoblastic leukemia without cranial irradiation: St Jude Total Therapy Study 16. J Clin Oncol. 2019;37:3377-91.

11. Takahashi H, Kajiwara R, Kato M, Hasegawa D, Tomizawa D, Noguchi Y, et al. Treatment outcome of children with acute lymphoblastic leukemia: the Tokyo Children’s Cancer Study Group (TCCSG) Study L04-16. Int J Hematol. 2018;108:98-108.

12. Yu CH, Jou ST, Su YH, Coustan-Smith E, Wu G, Cheng CN, et al. Clinical impact of minimal residual disease and genetic subtypes on the prognosis of childhood acute lymphoblastic leukemia. Cancer (in press).

13. Moorman AV, Antony G, Wade R, Butler ER, Enshaei A, Harrison CJ, et al. Time to cure for childhood and young adult acute lymphoblastic leukemia is independent of early risk factors: Long-term follow-up of the UKALL2003 trial. J Clin Oncol. 2022;40:4228-39.

14. Moorman AV, Barretta E, Butler ER, Ward EJ, Twentyman K, Kirkwood AA, et al. Prognostic impact of chromosomal abnormalities and copy number alterations in adult B-cell precursor acute lymphoblastic leukaemia: a UKALL14 study. Leukemia. 2022;36:625-36.

**Supplementary Table 7** Outcome of children with *ETV6*::*RUNX1*-positive BCP ALL in the NOPHO ALL2008 trial and in the literature

| Trial | MRD- | Age | 5-year^a^ survival (SE or CI) | | | | Reference |
| --- | --- | --- | --- | --- | --- | --- | --- |
|  | based | group | pCIR | pDFS | pEFS | pOS |  |
|  |  | (years) |  |  |  |  |  |
| NOPHO ALL2008 | Yes | 1-9 | 0.05 (0.01) | 0.92 (0.02) | 0.92 (0.02) | 0.98 (0.01) | Present |
| NOPHO ALL2008 | Yes | 10-17 | 0.20 (0.10) | 0.80 (0.10) | 0.88 (0.08) | 0.92 (0.07) | Present |
| NOPHO ALL2000 | No | 1-15 | NR | NR | 0.86 (0.03) | 0.96 (0.02) | 1 |
| NOPHO ALL1992 | No | 1-15 | NR | NR | 0.80 (0.03) | 0.88 (0.03)^b^ | 2 |
| AIEOP-BFM ALL | Yes | 1-18 | NR | NR | 0.949 (0.012) | NR | 3 |
| 2000 (SR) |  |  |  |  |  |  |  |
| AIEOP-BFM ALL | Yes | 1-18 | NR | NR | 0.817 (0.028) | NR | 3 |
| 2000 (IR) |  |  |  |  |  |  |  |
| AIEOP-BFM ALL | Yes | 1-18 | NR | NR | 0.549 (0.172) | NR | 3 |
| 2000 (HR) |  |  |  |  |  |  |  |
| CoALL 07-03 | Yes | 1-18 | NR | NR | 0.924 (0.02) | 0.982 (0.01) | 4 |
| COG AALL0331 | Yes | 1-9 | NR | NT | NR | 0.982 (0.004)^b^ | 5 |
| DCOG ALL10 | Yes | 1-18 | 0.049 (0.017) | NR | 0.951 (0.017) | 0.987 (0.009) | 6 |
| DFCI 05-001 | Yes | 1-18 | NR | NR | 0.95 (0.90-0.98) | NR | 7 |
| EORTC-CLG 58881 | No | 1-17 | NR | NR | 0.832 (0.036) | 0.933 (0.025) | 8 |
| EORTC-CLG 58951 | No | 1-17 | NR | NR | 0.908 (0.017) | 0.953 (0.013) | 8 |
| MRC ALL97/99 | No | 1-18 | NR | NR | 0.89 (0.85-0.91) | 0.96 (0.94-0.98) | 9 |
| St Jude Total | Yes | <18 | NR | NR | 0.984 (0.957-1.00) | 0.992 (0.972-1.00) | 10 |
| Therapy Study 16 |  |  |  |  |  |  |  |
| TCCSG L04-16 | No | 1-17 | NR | NR | 0.860 (0.027) | 0.974 (0.012) | 11 |
| TPOG-ALL-2013 | Yes | <18 | 0.029 (0.002-0.129) | NR | 0.914 (0.784-0.967) | 0.969 (0.798-0.996) | 12 |
| UKALL2003 | Yes | 1-24 | NR | NR | 0.914 (0.891-0.932)^c^ | 0.959 (0.942-0.971)^c^ | 13 |

BCP ALL, B-cell precursor acute lymphoblastic leukemia; CI, confidence interval; HR, high risk; IR, intermediate risk; MRD, measurable residual disease;

NR, not reported; pCIR, probability of cumulative incidence of relapse; pDFS, probability of disease-free survival; pEFS, probability of event-free survival; pOS, probability of overall survival; SE, standard error; SR, standard risk.

^a^Unless otherwise specified.

^b^At 6 yeats.

^c^At 10 years.

**References**

1. Schmiegelow K, Forestier E, Hellebostad M, Heyman M, Kristinsson J, Söderhäll S, et al. Long-term results of NOPHO ALL-92 and ALL-2000 studies of childhood acute lymphoblastic leukemia. Leukemia. 2010;24:345-54.

2. Forestier E, Heyman M, Andersen MK, Autio K, Blennow E, Borgström G, et al. Outcome of *ETV6*/*RUNX1*-positive childhood acute lymphoblastic leukaemia in the NOPHO-ALL-1992 protocol: frequent late relapses but good overall survival. Br J Haematol. 2008;140:665-72.

3. Conter V, Bartram CR, Valsecchi MG, Schrauder A, Panzer-Grümayer R, Möricke A, et al. Molecular response to treatment redefines all prognostic factors in children and adolescents with B-cell precursor acute lymphoblastic leukemia: results in 3184 patients of the AIEOP-BFM ALL 2000 study. Blood. 2010;115:3206-14.

4. Schramm F, zur Stadt U, Zimmermann M, Jorch N, Pekrun A, Borkhardt A, et al. Results of CoALL 07-03 study childhood ALL based on combined risk assessment by in vivo and in vitro pharmacosensitivity. Blood Adv. 2019;3:3688-99.

5. Mattano LA Jr, Devidas M, Maloney KW, Wang C, Friedmann AM, Buckley P, et al. Favorable trisomies and *ETV6*-*RUNX1* predict cure in low-risk B-cell acute lymphoblastic leukemia: Results from Children's Oncology Group trial AALL0331. J Clin Oncol. 2021;39:1540-52.

6. Pieters R, de Groot-Kruseman H, Van der Velden V, Fiocco M, van den Berg H, de Bont E, et al. Successful therapy reduction and intensification for childhood acute lymphoblastic leukemia based on minimal residual disease monitoring: study ALL10 from the Dutch Childhood Oncology Group. J Clin Oncol. 2016;34:2591-601.

7. Vrooman LM, Blonquist TM, Harris MH, Stevenson KE, Place AE, Hunt SK, et al. Refining risk classification in childhood B acute lymphoblastic leukemia: results of DFCI ALL Consortium Protocol 05-001. Blood Adv. 2018;2:1449-58.

8. Piette C, Suciu S, Clappier E, Bertrand Y, Drunat S, Girard S, et al. Differential impact of drugs on the outcome of *ETV6*-*RUNX1* positive childhood B-cell precursor acute lymphoblastic leukaemia: results of the EORTC CLG 58881 and 58951 trials. Leukemia. 2018;32:244-8.

9. Moorman AV, Ensor HM, Richards SM, Chilton L, Schwab C, Kinsey SE, et al. Prognostic effect of chromosomal abnormalities in childhood B-cell precursor acute lymphoblastic leukaemia: results from the UK Medical Research Council ALL97/99 randomised trial. Lancet Oncol. 2010;11:429-38.

10. Jeha S, Pei D, Choi J, Cheng C, Sandlund JT, Coustan-Smith E, et al. Improved CNS control of childhood acute lymphoblastic leukemia without cranial irradiation: St Jude Total Therapy Study 16. J Clin Oncol. 2019;37:3377-91.

11. Takahashi H, Kajiwara R, Kato M, Hasegawa D, Tomizawa D, Noguchi Y, et al. Treatment outcome of children with acute lymphoblastic leukemia: the Tokyo Children’s Cancer Study Group (TCCSG) Study L04-16. Int J Hematol. 2018;108:98-108.

12. Yu CH, Jou ST, Su YH, Coustan-Smith E, Wu G, Cheng CN, et al. Clinical impact of minimal residual disease and genetic subtypes on the prognosis of childhood acute lymphoblastic leukemia. Cancer (in press).

13. Moorman AV, Antony G, Wade R, Butler ER, Enshaei A, Harrison CJ, et al. Time to cure for childhood and young adult acute lymphoblastic leukemia is independent of early risk factors: Long-term follow-up of the UKALL2003 trial. J Clin Oncol. 2022;40:4228-39.

**Supplementary Table 8** Outcome of children and adults with *TCF3*::*PBX1*-positive BCP ALL in the NOPHO ALL2008 trial and in the literature

| Trial | MRD- | Age | | 5-year^a^ survival (SE or CI) | | | | Reference |  |
| --- | --- | --- | --- | --- | --- | --- | --- | --- | --- |
|  | based | group | pCIR | | pDFS | pEFS | pOS |  |  |
|  |  | (years) |  | |  |  |  |  |  |
| NOPHO ALL2008 | Yes | 1-45 | 0.02 (0.02) | | 0.96 (0.03) | 0.96 (0.03) | 0.98 (0.02) | Present |  |
| NOPHO ALL2008 | Yes | 1-9 | 0.03 (0.03) | | 0.97 (0.03) | 0.97 (0.03) | 1.0 | Present |  |
| NOPHO ALL2008 | Yes | 10-17 | 0 | | 0.89 (0.10) | 0.89 (0.10) | 0.89 (0.10) | Present |  |
| NOPHO ALL2008 | Yes | 18-45 | 0 | | 1.0 | 1.0 | 1.0 | Present |  |
| NOPHO ALL2000 | No | 1-15 | NR | | NR | 0.82 (0.08) | 0.87 (0.07) | 1 |  |
| CCLSG ALL 2004 | No | 1-18 | NR | | NR | 0.828 (0.070) | 0.863 (0.064) | 2 |  |
| DCOG ALL10 | Yes | 1-18 | 1.0 | | NR | 1.0 | 1.0 | 3 |  |
| DFCI 05-001 | Yes | 1-18 | NR | | NR | 0.82 (0.59-0.93) | NR | 4 |  |
| GIMEMA 0496 | No | 16-60 | NR | | 0.43 (0.062-0.795) | NR | NR | 5 |  |
| GRAALL 2003/2005 | Partly | 15-59 | NR | | NR | 0.536 (0.34-0.70) | 0.571 (0.37-0.73) | 6 |  |
| JACLS ALL02 | No | 1-18 | NR | | NR | 0.854 (0.039)^b^ | 0.890 (0.03)^b^ | 2 |  |
| MRC ALL97/99 | No | 1-18 | NR | | NR | 0.80 (0.66-0.89) | 0.84 (0.71-0.92) | 7 |  |
| MRC UKALLXII/ | No | 15-65 | NR | | NR | 0.29 (0.13-0.48) | 0.32 (0.14-0.51) | 8 |  |
| ECOG 2993 |  |  |  | |  |  |  |  |  |
| St Jude Total | Yes | <18 | NR | | NR | 0.871 (0.695-0.100) | 0.871 (0.695-0.100) | 9 |  |
| Therapy Study 16 |  |  |  | |  |  |  |  |  |
| TCCSG L04-16 | No | 1-17 | NR | | NR | 0.907 (0.036) | 0.969 (0.021) | 10 |  |
| TPOG-ALL-2013 | Yes | <18 | 0.100 (0.05-0.374) | | NR | 0.800 (0.409-0.946) | 0.800 (0.409-0.946) | 11 |  |
| UKALL2003 | Yes | 1-24 | NR | | NR | 0.898 (0.813-0.945)^c^ | 0.920 (0.840-0.961)^c^ | 12 |  |
| UKALL14 | Yes | 25-65 | NR | | NR | 0.49 (0.22-0.72)^b^ | 0.54 (0.25-0.77)^b^ | 13 |  |

BCP ALL, B-cell precursor acute lymphoblastic leukemia; CI, confidence interval; MRD, measurable residual disease; NR, not reported; pCIR, probability of

cumulative incidence of relapse; pDFS, probability of disease-free survival; pEFS, probability of event-free survival; pOS, probability of overall survival; SE, standard error.

^a^Unless otherwise specified.

^b^At 3 years.

^c^At 10 years.

**References**

1. Schmiegelow K, Forestier E, Hellebostad M, Heyman M, Kristinsson J, Söderhäll S, et al. Long-term results of NOPHO ALL-92 and ALL-2000 studies of childhood acute lymphoblastic leukemia. Leukemia. 2010;24:345-54.

2. Asai D, Imamura T, Yamashita Y, Suenobu S-i, Moriya-Saito A, Hasegawa D, et al. Outcome of *TCF3*-*PBX1* positive pediatric acute lymphoblastic leukemia patients in Japan: a collaborative study of Japan Association of Childhood Leukemia Study (JACLS) and Children's Cancer and Leukemia Study Group (CCLSG). Cancer Med. 2014;3:623-31.

3. Pieters R, de Groot-Kruseman H, Van der Velden V, Fiocco M, van den Berg H, de Bont E, et al. Successful therapy reduction and intensification for childhood acute lymphoblastic leukemia based on minimal residual disease monitoring: study ALL10 from the Dutch Childhood Oncology Group. J Clin Oncol. 2016;34:2591-601.

4. Vrooman LM, Blonquist TM, Harris MH, Stevenson KE, Place AE, Hunt SK, et al. Refining risk classification in childhood B acute lymphoblastic leukemia: results of DFCI ALL Consortium Protocol 05-001. Blood Adv. 2018;2:1449-58.

5. Mancini M, Scappaticci D, Cimino G, Nanni M, Derme V, Elia L, et al. A comprehensive genetic classification of adult acute lymphoblastic leukemia (ALL): analysis of the GIMEMA 0496 protocol. Blood. 2005;105:3434-41.

6. Lafage-Pochitaloff M, Baranger L, Hunault M, Cuccuini W, Lefebvre C, Bidet A, et al. Impact of cytogenetic abnormalities in adults with Ph-negative B-cell precursor acute lymphoblastic leukemia. Blood. 2017;130:1832-44.

7. Moorman AV, Ensor HM, Richards SM, Chilton L, Schwab C, Kinsey SE, et al. Prognostic effect of chromosomal abnormalities in childhood B-cell precursor acute lymphoblastic leukaemia: results from the UK Medical Research Council ALL97/99 randomised trial. Lancet Oncol. 2010;11:429-38.

8. Moorman AV, Harrison CJ, Buck GAN, Richards SM, Secker-Walker LM, Martineau M, et al. Karyotype is an independent prognostic factor in adult acute lymphoblastic leukemia (ALL): analysis of cytogenetic data from patients treated on the Medical Research Council (MRC) UKALLXII/Eastern Cooperative Oncology Group (ECOG) 2993 trial. Blood. 2007;109:3189-97.

9. Jeha S, Pei D, Choi J, Cheng C, Sandlund JT, Coustan-Smith E, et al. Improved CNS control of childhood acute lymphoblastic leukemia without cranial irradiation: St Jude Total Therapy Study 16. J Clin Oncol. 2019;37:3377-91.

10. Takahashi H, Kajiwara R, Kato M, Hasegawa D, Tomizawa D, Noguchi Y, et al. Treatment outcome of children with acute lymphoblastic leukemia: the Tokyo Children’s Cancer Study Group (TCCSG) Study L04-16. Int J Hematol. 2018;108:98-108.

11. Yu CH, Jou ST, Su YH, Coustan-Smith E, Wu G, Cheng CN, et al. Clinical impact of minimal residual disease and genetic subtypes on the prognosis of childhood acute lymphoblastic leukemia. Cancer (in press).

12 Moorman AV, Antony G, Wade R, Butler ER, Enshaei A, Harrison CJ, et al. Time to cure for childhood and young adult acute lymphoblastic leukemia is independent of early risk factors: Long-term follow-up of the UKALL2003 trial. J Clin Oncol. 2022;40:4228-39.

13. Moorman AV, Barretta E, Butler ER, Ward EJ, Twentyman K, Kirkwood AA, et al. Prognostic impact of chromosomal abnormalities and copy number alterations in adult B-cell precursor acute lymphoblastic leukaemia: a UKALL14 study. Leukemia. 2022;36:625-36.

**Supplementary Table 9** The distributions of genetic subtypes and types/frequencies of events in the final BCP ALL risk stratification groups^a^

| Genetic subtype | Type of | SR | IR | HR-chemo | HR-SCT |
| --- | --- | --- | --- | --- | --- |
|  | event | *n* = 761 | *n* = 520 | *n* = 99 | *n* = 95 |
| HeH |  | *n* = 301 | *n* = 144 | *n* = 7 | *n* = 13 |
| (*n* = 465) | Deaths | 6 | 6 | 1 | 1 |
|  | Relapse | 11 | 14 | 1 | 1 |
|  | DCR1 | 3 | 2 | 0 | 1 |
|  | SMN | 6 | 0 | 0 | 0 |
| *ETV6*::*RUNX1* |  | *n* = 240 | *n* = 99 | *n* = 2 | *n* = 4 |
| (*n* = 345) | Deaths | 5 | 4 | 0 | 2 |
|  | Relapse | 10 | 11 | 0 | 2 |
|  | DCR1 | 2 | 3 | 0 | 0 |
|  | SMN | 2 | 1 | 0 | 0 |
| *TCF3*::*PBX1* |  | *n* = 0 | *n* = 47 | *n* = 0 | *n* = 1 |
| (*n* = 48) | Deaths | - | 1 | - | 0 |
|  | Relapse | - | 1 | - | 0 |
|  | DCR1 | - | 1 | - | 0 |
|  | SMN | - | 0 | - | 0 |
| *KMT2A*-r |  | *n* = 0 | *n* = 0 | *n* = 44 | *n* = 3 |
| (*n* = 47) | Deaths | - | - | 10 | 2 |
|  | Relapse | - | - | 6 | 3 |
|  | DCR1 | - | - | 4 | 0 |
|  | SMN | - | - | 1 | 0 |
| dic(9;20) |  | *n* = 0 | *n* = 27 | *n* = 2 | *n* = 0 |
| (*n* = 29) | Deaths | . | 1 | 0 | - |
|  | Relapse | - | 2 | 2 | - |
|  | DCR1 | - | 1 | 0 | - |
|  | SMN | - | 0 | 0 | - |
| iAMP21 |  | *n* = 0 | *n* = 23 | *n* = 0 | *n* = 4 |
| (*n* = 27) | Deaths | - | 3 | - | 1 |
|  | Relapse | - | 9 | - | 0 |
|  | DCR1 | - | 0 | - | 1 |
|  | SMN | - | 0 | - | 0 |
| HoL |  | *n* = 0 | *n* = 0 | *n* =14 | *n* = 1 |
| (*n* = 15) | Deaths | - | - | 4 | 1 |
|  | Relapse | - | - | 4 | 1 |
|  | DCR1 | - | - | 0 | 0 |
|  | SMN | - | - | 2 | 0 |
| NH |  | *n* = 0 | *n* = 0 | *n* = 8 | *n* = 0 |
| (*n* = 8) | Deaths | - | - | 4 | - |
|  | Relapse | - | - | 1 | - |
|  | DCR1 | - | - | 3 | - |
|  | SMN | - | - | 0 | - |
| B-other |  | *n* = 220 | *n* = 120 | *n* = 17 | *n* = 61 |
| (*n* = 418) | Deaths | 18 | 20 | 8 | 16 |
|  | Relapse | 30 | 30 | 7 | 15 |
|  | DCR1 | 4 | 2 | 2 | 5 |
|  | SMN | 1 | 0 | 0 | 0 |
| Unknown |  | *n* = 0 | *n* = 60 | *n* = 5 | *n* = 8 |
| (*n* = 73) | Deaths | - | 4 | 0 | 2 |
|  | Relapse | - | 11 | 2 | 2 |
|  | DCR1 | - | 2 | 0 | 0 |
|  | SMN | - | 0 | 0 | 0 |

BCP ALL, B-cell precursor acute lymphoblastic leukemia; HeH, high hyperdiploidy (51-67

chromosomes); DCR1, death in complete remission; HoL, low hypodiploidy (30-39 chromosomes); HR-chemo, high-risk patients stratified to treatment with chemotherapy only; HR-SCT, high-risk patients stratified to stem cell transplantation; iAMP21, intrachromosomal amplification of chromosome 21; IR, intermediate risk; *KMT2A*-r, *KMT2A* rearrangement; *n*, number of patients; NH, near-haploidy (24-29 chromosomes); SMN, second malignant neoplasm; SR, standard risk.

^a^Eighteen patients had no final risk group: 14 with induction death, one lost to follow-up, one leaving protocol therapy before EOI, and two receiving such a modified therapy that they were considered outliers

**Supplementary Table 10** Outcome of children and adults with dic(9;20)(p13;q11)-positive BCP ALL in the NOPHO ALL2008 trial and in the literature

| Trial | MRD- | Age | 5-year survival (SE or CI) | | | | Reference |  |
| --- | --- | --- | --- | --- | --- | --- | --- | --- |
|  | based | group | pCIR | pDFS | pEFS | pOS |  |  |
|  |  | (years) |  |  |  |  |  |  |
| NOPHO ALL2008 | Yes | 1-45 | 0.14 (0.06) | 0.83 (0.07) | 0.83 (0.07) | 0.97 (0.03) | Present |  |
| NOPHO ALL2008 | Yes | 1-9 | 0.15 (0.07) | 0.81 (0.08) | 0.81 (0.08) | 0.96 (0.04) | Present |  |
| NOPHO ALL2008 | Yes | 10-17 | 0 | 1.0 | 1.0 | 1.0 | Present |  |
| NOPHO ALL2008 | Yes | 18-45 | 0 | 1.0 | 1.0 | 1.0 | Present |  |
| NOPHO ALL2000 | No | 1-15 | NR | NR | 0.69 (0.09) | 0.85 (0.07) | 1 |  |
| BFM ALL 2000 | Yes | 1-18 | NR | NR | 0.75 (0.11) | 0.94 (0.06) | 2 |  |
| MRC ALL97/99 | No | 1-18 | NR | NR | 0.77 (0.44-0.92) | 0.92 (0.57-0.99) | 3 |  |

BCP ALL, B-cell precursor acute lymphoblastic leukemia; CI, confidence interval; MRD, measurable residual disease; NR, not reported; pCIR, probability of

cumulative incidence of relapse; pDFS, probability of disease-free survival; pEFS, probability of event-free survival; pOS, probability of overall survival; SE, standard error.

**References**

1. Zachariadis V, Gauffin F, Kuchinskaya E, Heyman M, Schoumans J, Blennow E, et al. The frequency and prognostic impact of dic(9;20)(p13.2;q11.2) in childhood B-cell precursor acute lymphoblastic leukemia: results from the NOPHO ALL-2000 trial. Leukemia. 2011;25:622-8.

2. Pichler H, Möricke A, Mann G, Teigler-Schlegel A, Niggli F, Nebral K, et al. Prognostic relevance of dic(9;20)(p11;q13) in childhood B-cell precursor acute lymphoblastic leukaemia treated with Berlin-Frankfurt-Münster (BFM) protocols containing an intensive induction and post-induction consolidation therapy. Br J Haematol. 2010;149:93-100.

3. Moorman AV, Ensor HM, Richards SM, Chilton L, Schwab C, Kinsey SE, et al. Prognostic effect of chromosomal abnormalities in childhood B-cell precursor acute lymphoblastic leukaemia: results from the UK Medical Research Council ALL97/99 randomised trial. Lancet Oncol. 2010;11:429-38.

**Supplementary Table 11** Outcome of children and adults with iAMP21-positive BCP ALL in the NOPHO ALL2008 trial and in the literature

| Trial | MRD- | Age | 5-year^a^ survival (SE or CI) | | | | Reference |  |
| --- | --- | --- | --- | --- | --- | --- | --- | --- |
|  | based | group | pCIR | pDFS | pEFS | pOS |  |  |
|  |  | (years) |  |  |  |  |  |  |
| NOPHO ALL2008 | Yes | 1-45 | 0.31 (0.09) | 0.66 (0.09) | 0.66 (0.09) | 0.92 (0.05) | Present |  |
| NOPHO ALL2008 | Yes | 1-9 | 0.40 (0.14) | 0.60 (0.14) | 0.60 (0.14) | 0.92 (0.08) | Present |  |
| NOPHO ALL2008 | Yes | 10-17 | 0.25 (0.12) | 0.67 (0.14) | 0.67 (0.14) | 0.92 (0.08) | Present |  |
| NOPHO ALL2000 | No | 1-15 | NR | NR | 0.50 (0.14) | 0.81 (0.12) | 1 |  |
| ALL-BFM 86/90/95/2000 | Partly | <18 | NR | NR | 0.37 (0.14)^b^ | 0.66 (0.11)^b^ | 2 |  |
| COG AALL0331/0232 | Yes | 1-30 | NR | NR | 0.727 (0.058)^c^ | 0.876 (0.044)^c^ | 3 |  |
| DFCI 05-001 | Yes | 1-18 | NR | NR | 0.67 (0.33-0.86) | NR | 4 |  |
| MRC ALL97/99 | No | 1-18 | NR | NR | 0.29 (0.14-0.46) | 0.67 (0.47-0.82) | 5 |  |
| Ponte di Legno | Partly | 2-30 | NR | NR | 0.58 (0.51-0.65) | 0.82 (0.76-0.87) | 6 |  |
| Study Group |  |  |  |  |  |  |  |  |
| UKALL2003 | Yes | 1-24 | NR | NR | 0.741 (0.602-0.833)^d^ | 0.849 (0.720-0.922)^d^ | 7 |  |

BCP ALL, B-cell precursor acute lymphoblastic leukemia; CI, confidence interval; iAMP21, intrachromosomal amplification of chromosome 21; MRD,

measurable residual disease; NR, not reported; pCIR, probability of cumulative incidence of relapse; pDFS, probability of disease-free survival; pEFS, probability of event-free survival; pOS, probability of overall survival; SE, standard error.

^a^Unless otherwise specified.

^b^At 6 years

^c^At 4 years.

^d^At 10 years.

**References**

1. Zachariadis V, Gauffin F, Kuchinskaya E, Heyman M, Schoumans J, Blennow E, et al. The frequency and prognostic impact of dic(9;20)(p13.2;q11.2) in childhood B-cell precursor acute lymphoblastic leukemia: results from the NOPHO ALL-2000 trial. Leukemia. 2011;25:622-8.

2. Attarbaschi A, Mann G, Panzer-Grümayer R, Röttgers S, Steiner M, König M, et al. Minimal residual disease values discriminate between low and high relapse risk in children with B-cell precursor acute lymphoblastic leukemia and an intrachromosomal amplification of chromosome 21: the Austrian and German acute lymphoblastic leukemia Berlin-Frankfurt-Munster (ALL-BFM) trials. J Clin Oncol. 2008;26:3046-50.

3. Heerema NA, Carroll AJ, Devidas M, Loh ML, Borowitz MJ, Gastier-Foster JM, et al. Intrachromosomal amplification of chromosome 21 is associated with inferior outcomes in children with acute lymphoblastic leukemia treated in contemporary standard-risk children's oncology group studies: a report from the children's oncology group. J Clin. Oncol. 2013;31:3397-402.

4. Vrooman LM, Blonquist TM, Harris MH, Stevenson KE, Place AE, Hunt SK, et al. Refining risk classification in childhood B acute lymphoblastic leukemia: results of DFCI ALL Consortium Protocol 05-001. Blood Adv. 2018;2:1449-58.

5. Moorman AV, Robinson H, Schwab C, Richards SM, Hancock J, Mitchell CD, et al. Risk-directed treatment intensification significantly reduces the risk of relapse among children and adolescents with acute lymphoblastic leukemia and intrachromosomal amplification of chromosome 21: a comparison of the MRC ALL97/99 and UKALL2003 trials. J Clin Oncol. 2013;31:3389-96.

6. Harrison CJ, Moorman AV, Schwab C, Carroll AJ, Raetz EA, Devidas M, et al. An international study of intrachromosomal amplification of chromosome 21 (iAMP21): cytogenetic characterization and outcome. Leukemia. 2014;28:1015-21.

7 Moorman AV, Antony G, Wade R, Butler ER, Enshaei A, Harrison CJ, et al. Time to cure for childhood and young adult acute lymphoblastic leukemia is independent of early risk factors: Long-term follow-up of the UKALL2003 trial. J Clin Oncol. 2022;40:4228-39.

**Supplementary Table 12** Outcome of children and adults with *KMT2A*-rearranged BCP ALL in the NOPHO ALL2008 trial and in the literature

| Trial | MRD- | Age | 5-year^a^ survival (SE or CI) | | | | Reference |  |
| --- | --- | --- | --- | --- | --- | --- | --- | --- |
|  | based | group | pCIR | pDFS | pEFS | pOS |  |  |
|  |  | (years) |  |  |  |  |  |  |
| NOPHO ALL2008 | Yes | 1-45 | 0.19 (0.06) | 0.70 (0.07) | 0.65 (0.07) | 0.69 (0.07) | Present |  |
| NOPHO ALL2008 | Yes | 1-9 | 0.08 (0.06) | 0.83 (0.08) | 0.77 (0.08) | 0.81 (0.08) | Present |  |
| NOPHO ALL2008 | Yes | 10-17 | 0.12 (0.12) | 0.75 (0.15) | 0.75 (0.15) | 0.88 (0.12) | Present |  |
| NOPHO ALL2008 | Yes | 18-45 | 0.40 (0.13) | 0.47 (0.13) | 0.41 (0.12) | 0.41 (0.12) | Present |  |
| NOPHO ALL2000 | No | 1-15 | NR | NR | 0.56 (0.12) | 0.61 (0.11) | 1 |  |
| DFCI 05-001 | Yes | 1-18 | NR | NR | 0.58 (0.27-0.80) | NR | 2 |  |
| GIMEMA 0496 [t(4;11)] | No | 16-60 | NR | 0.48 (0.263-0.690) | NR | NR | 3 |  |
| GRAALL 2003/ | Partly | 15-59 | NR | NR | 0.428 (0.31-0.54) | 0.457 (0.34-0.57) | 4 |  |
| 2005 [t(4;11] |  |  |  |  |  |  |  |  |
| GRAALL 2003/ | Partly | 15-59 | NR | NR | 0.636 (0.30-0.85) | 0.727 (0.37-0.90) | 4 |  |
| 2005 [other 11q23] |  |  |  |  |  |  |  |  |
| MRC ALL97/99 | No | 1-18 | NR | NR | 0.50 (0.31-0.66) | 0.60 (0.41-0.75) | 5 |  |
| MRC UKALLXII/ | No | 15-65 | NR | NR | 0.24 (0.13-0.36) | 0.24 (0.13-0.36) | 6 |  |
| ECOG 2993 [t(4;11] |  |  |  |  |  |  |  |  |
| MRC UKALLXII/ | No | 15-65 | NR | NR | 0.29 (0.09-0.52) | 0.33 (0.12-0.56) | 6 |  |
| ECOG 2993 [other 11q23] |  |  |  |  |  |  |  |  |
| St Jude Total | Yes | <18 | NR | NR | 0.649 (0.461-0.837) | 0.789 (0.617-0.961) | 7 |  |
| Therapy Study 16 |  |  |  |  |  |  |  |  |
| TCCSG L04-16 | No | 1-17 | NR | NR | 0.603 (0.109) | 0.790 (0.094) | 8 |  |
| TPOG-ALL-2013 | Yes | <18 | 0.314 (0.030-0.683) | NR | 0.536 (0.132-0.825) | 0.643 (0.151-0.902) | 9 |  |
| UKALL2003 | Yes | 1-24 | NR | NR | 0.691 (0.527-0.807)^b^ | 0.738 (0.577-0.846)^b^ | 10 |  |
| UKALL14 [t(4;11)] | Yes | 25-65 | NR | NR | 0.38 (0.25-0.52)^c^ | 0.46 (0.31-0.59)^c^ | 11 |  |
| UKALL14 [other 11q23] | Yes | 25-65 | NR | NR | 0.33 (0.08-0.62)^c^ | 0.44 (0.14-0.72)^c^ | 11 |  |

BCP ALL, B-cell precursor acute lymphoblastic leukemia; CI, confidence interval; MRD, measurable residual disease; NR, not reported; pCIR, probability of

cumulative incidence of relapse; pDFS, probability of disease-free survival; pEFS, probability of event-free survival; pOS, probability of overall survival; SE, standard error.

^a^Unless otherwise specified.

^b^At 10 years

^c^At 3 years.

**References**

1. Zachariadis V, Gauffin F, Kuchinskaya E, Heyman M, Schoumans J, Blennow E, et al. The frequency and prognostic impact of dic(9;20)(p13.2;q11.2) in childhood B-cell precursor acute lymphoblastic leukemia: results from the NOPHO ALL-2000 trial. Leukemia. 2011;25:622-8.

2. Vrooman LM, Blonquist TM, Harris MH, Stevenson KE, Place AE, Hunt SK, et al. Refining risk classification in childhood B acute lymphoblastic leukemia: results of DFCI ALL Consortium Protocol 05-001. Blood Adv. 2018;2:1449-58.

3. Mancini M, Scappaticci D, Cimino G, Nanni M, Derme V, Elia L, et al. A comprehensive genetic classification of adult acute lymphoblastic leukemia (ALL): analysis of the GIMEMA 0496 protocol. Blood. 2005;105:3434-41.

4. Lafage-Pochitaloff M, Baranger L, Hunault M, Cuccuini W, Lefebvre C, Bidet A, et al. Impact of cytogenetic abnormalities in adults with Ph-negative B-cell precursor acute lymphoblastic leukemia. Blood. 2017;130:1832-44.

5. Moorman AV, Ensor HM, Richards SM, Chilton L, Schwab C, Kinsey SE, et al. Prognostic effect of chromosomal abnormalities in childhood B-cell precursor acute lymphoblastic leukaemia: results from the UK Medical Research Council ALL97/99 randomised trial. Lancet Oncol. 2010;11:429-38.

6. Moorman AV, Harrison CJ, Buck GAN, Richards SM, Secker-Walker LM, Martineau M, et al. Karyotype is an independent prognostic factor in adult acute lymphoblastic leukemia (ALL): analysis of cytogenetic data from patients treated on the Medical Research Council (MRC) UKALLXII/Eastern Cooperative Oncology Group (ECOG) 2993 trial. Blood. 2007;109:3189-97.

7. Jeha S, Pei D, Choi J, Cheng C, Sandlund JT, Coustan-Smith E, et al. Improved CNS control of childhood acute lymphoblastic leukemia without cranial irradiation: St Jude Total Therapy Study 16. J Clin Oncol. 2019;37:3377-91.

8. Takahashi H, Kajiwara R, Kato M, Hasegawa D, Tomizawa D, Noguchi Y, et al. Treatment outcome of children with acute lymphoblastic leukemia: the Tokyo Children’s Cancer Study Group (TCCSG) Study L04-16. Int J Hematol. 2018;108:98-108.

9. Yu CH, Jou ST, Su YH, Coustan-Smith E, Wu G, Cheng CN, et al. Clinical impact of minimal residual disease and genetic subtypes on the prognosis of childhood acute lymphoblastic leukemia. Cancer (in press).

10 Moorman AV, Antony G, Wade R, Butler ER, Enshaei A, Harrison CJ, et al. Time to cure for childhood and young adult acute lymphoblastic leukemia is independent of early risk factors: Long-term follow-up of the UKALL2003 trial. J Clin Oncol. 2022;40:4228-39.

11. Moorman AV, Barretta E, Butler ER, Ward EJ, Twentyman K, Kirkwood AA, et al. Prognostic impact of chromosomal abnormalities and copy number alterations in adult B-cell precursor acute lymphoblastic leukaemia: a UKALL14 study. Leukemia. 2022;36:625-36.

**Supplementary Table 13** Outcome of pediatric and adult low hypodiploid BCP ALL in the NOPHO ALL2008 trial and in the literature

| Trial | MRD- | Age | 5-year^a^ survival (SE or CI) | | | | Reference |  |
| --- | --- | --- | --- | --- | --- | --- | --- | --- |
|  | based | group | pCIR | pDFS | pEFS | pOS |  |  |
|  |  | (years) |  |  |  |  |  |  |
| NOPHO ALL2008 | Yes | 1-45 | 0.27 (0.11) | 0.60 (0.13) | 0.60 (0.13) | 0.67 (0.12) | Present |  |
| NOPHO ALL2008 | Yes | 1-9 | 0.33 (0.27) | 0.67 (0.27) | 0.67 (0.27) | 0.67 (0.27) | Present |  |
| NOPHO ALL2008 | Yes | 10-17 | 0.14 (0.13) | 0.57 (0.19) | 0.57 (0.19) | 0.57 (0.19) | Present |  |
| NOPHO ALL2008 | Yes | 18-45 | 0.40 (0.22) | 0.60 (0.22) | 0.60 (0.22) | 0.80 (0.18) | Present |  |
| GRAALL 2003/2005 | Partly | 15-59 | NR | NR | 0.358 (0.20-0.52) | 0.386 (0.22-0.55) | 1 |  |
| MRC ALL97/99 | No | 1-18 | NR | NR | 0.50 (0.15-0.78) | 0.50 (0.15-0.78) | 2 |  |
| MRC UKALLXII/ | No | 15-65 | NR | NR | 0.18 (0.07-0.34) | 0.22 (0.09-0.38) | 3 |  |
| ECOG 2993 |  |  |  |  |  |  |  |  |
| Multinational Study^b^ | Partly | <21 | NR | NR | 0.57 (0.48-0.67) | 0.62 (0.54-0.72) | 4 |  |
| St. Jude Total Therapy | Yes | 1-18 | NR | NR | 0.750 (0.408-0.912) | NR | 5 |  |
| Studis 15 and 16 |  |  |  |  |  |  |  |  |
| UKALL14 | Yes | 25-65 | NR | NR | 0.20 (0.10-0.33)^c^ | 0.22 (0.11-0.34)^c^ | 6 |  |

BCP ALL, B-cell precursor acute lymphoblastic leukemia; CI, confidence interval; MRD, measurable residual disease; NR, not reported; pCIR, probability of

cumulative incidence of relapse; pDFS, probability of disease-free survival; pEFS, probability of event-free survival; pOS, probability of overall survival; SE, standard error.

^a^Unless otherwise specified.

^b^Based on 16 cooperative study groups or single institutions.

^c^At 3 years.

**References**

1. Lafage-Pochitaloff M, Baranger L, Hunault M, Cuccuini W, Lefebvre C, Bidet A, et al. Impact of cytogenetic abnormalities in adults with Ph-negative B-cell precursor acute lymphoblastic leukemia. Blood. 2017;130:1832-44.

2. Moorman AV, Ensor HM, Richards SM, Chilton L, Schwab C, Kinsey SE, et al. Prognostic effect of chromosomal abnormalities in childhood B-cell precursor acute lymphoblastic leukaemia: results from the UK Medical Research Council ALL97/99 randomised trial. Lancet Oncol. 2010;11:429-38.

3. Moorman AV, Harrison CJ, Buck GAN, Richards SM, Secker-Walker LM, Martineau M, et al. Karyotype is an independent prognostic factor in adult acute lymphoblastic leukemia (ALL): analysis of cytogenetic data from patients treated on the Medical Research Council (MRC) UKALLXII/Eastern Cooperative Oncology Group (ECOG) 2993 trial. Blood. 2007;109:3189-97.

4. Pui C-H, Rebora P, Schrappe M, Attarbaschi A, Baruchel A, Basso G, et al. Outcome of children with hypodiploid acute lymphoblastic leukemia: a retrospective multinational study. J Clin Oncol. 2019;37:770-9.

5. Mullighan CG, Jeha S, Pei D, Payne-Turner D, Coustan-Smith E, Roberts KG, et al. Outcome of children with hypodiploid ALL treated with risk-directed therapy based on MRD levels. Blood. 2015;126:2896-9.

6. Moorman AV, Barretta E, Butler ER, Ward EJ, Twentyman K, Kirkwood AA, et al. Prognostic impact of chromosomal abnormalities and copy number alterations in adult B-cell precursor acute lymphoblastic leukaemia: a UKALL14 study. Leukemia. 2022;36:625-36.

**Supplementary Table 14** Outcome of pediatric near-haploid BCP ALL in the NOPHO ALL2008 trial and in the literature

| Trial | MRD- | Age | 5-year survival (SE or CI) | | | | Reference |  |
| --- | --- | --- | --- | --- | --- | --- | --- | --- |
|  | based | group | pCIR | pDFS | pEFS | pOS |  |  |
|  |  | (years) |  |  |  |  |  |  |
| NOPHO ALL2008 | Yes | 1-9 | 0.12 (0.12) | 0.50 (0.18) | 0.50 (0.18) | 0.50 (0.18) | Present |  |
| MRC ALL97/99 | No | 1-18 | NR | NR | 0.40 (0.12-0.67) | 0.40 (0.12-0.67) | 1 |  |
| Multinational Study^a^ | Partly | <21 | NR | NR | 0.45 (0.36-0.56) | 0.54 (0.45-0.65) | 2 |  |
| St. Jude Total Therapy | Yes | 1-18 | NR | NR | 0.729 (0.276-0.925) | NR | 3 |  |
| Studies 15 and 16 |  |  |  |  |  |  |  |  |

BCP ALL, B-cell precursor acute lymphoblastic leukemia; CI, confidence interval; MRD, measurable residual disease; NR, not reported; pCIR, probability of

cumulative incidence of relapse; pDFS, probability of disease-free survival; pEFS, probability of event-free survival; pOS, probability of overall survival; SE, standard error.

^a^Based on 16 cooperative study groups or single institutions.

**References**

1. Moorman AV, Ensor HM, Richards SM, Chilton L, Schwab C, Kinsey SE, et al. Prognostic effect of chromosomal abnormalities in childhood B-cell precursor acute lymphoblastic leukaemia: results from the UK Medical Research Council ALL97/99 randomised trial. Lancet Oncol. 2010;11:429-38.

2. Pui C-H, Rebora P, Schrappe M, Attarbaschi A, Baruchel A, Basso G, et al. Outcome of children with hypodiploid acute lymphoblastic leukemia: a retrospective multinational study. J Clin Oncol. 2019;37:770-9.

3. Mullighan CG, Jeha S, Pei D, Payne-Turner D, Coustan-Smith E, Roberts KG, et al. Outcome of children with hypodiploid ALL treated with risk-directed therapy based on MRD levels. Blood. 2015;126:2896-9.

**Supplementary Table 15** Final treatment administered to the BCP ALL patients finally stratified to SR and IR treatment according to intention-to-treat

| Final risk | Final treatment administered | | |  |
| --- | --- | --- | --- | --- |
| stratification | SR | IR | HR^a^ | Modified |
| group | *n* = 781 | *n* = 488 | *n* = 7 | *n* = 5 |
| SR (*n* = 761) | 742 (98%) | 17 (2.2%) | 2 (0.3%) | 0 |
| IR (*n* = 520) | 39 (7.5%) | 471 (91%) | 5 (1%) | 5 (1%) |

BCP ALL, B-cell precursor acute lymphoblastic leukemia; HR, HR-chemo, high-risk chemotherapy only or HR-SCT, stem cell transplantation; IR, intermediate risk; *n*, number of patients; SR, standard risk. Modified, treatment modified from the intended IR-stratification due to diverse toxicities in five patients.

^a^Seven patients intended for treatment according to SR (*n*=2) and IR (*n*=5), according to the protocol, were in the end treated according to HR-chemo or HR-SCT due to local clinical decisions based on various reasons.

**Supplementary Table 16** Cox regression analyses of the disease-free and overall survival of the 1281 BCP ALL patients^a^ finally stratified to standard and intermediate risk groups in the NOPHO ALL2008 trial

| Variables | Disease-free survival | | | | | |  | | | Overall survival | | | | | | |
| --- | --- | --- | --- | --- | --- | --- | --- | --- | --- | --- | --- | --- | --- | --- | --- | --- |
|  | Univariate | | Multivariable | | | | |  | | Univariate | | | | Multivariable | | |
|  | HR (95% CI) | *P* | HR (95% CI) | *P* | | | |  | HR (95% CI) | | | *P* | | HR (95% CI) | *P* | |
| Measurable residual disease |  |  |  |  | | | |  |  | | |  | |  |  | |
| Day 29 undetectable <0.1% (*n* = 526) | 1.0 |  | 1.0 | |  | | |  | 1.0 | | |  | | 1.0 |  | |
| Day 29 detectable <0.1% (*n* = 433) | 1.48 (0.9-2.2) | 0.059 | 1.44 (0.9-2.3) | | 0.132 | | |  | 1.22 (0.6-2.3) | | | 0.532 | | 1.14 (0.5-2.5) | 0.735 | |
| Day 29 ≥0.1% and ≤5% (*n* = 322) | **2.67 (1.8-3.9)** | **<0.001** | 1.42 (0.3-5.9) | | 0.632 | | |  | **2.71 (1.5-4.8)** | | | **<0.001** | | 2.42 (0.5-11) | 0.235 | |
| ^b^Final treatment administered |  |  |  | |  | | |  |  | | |  | |  |  | |
| Standard risk (*n* = 781) | 1.0 |  | 1.0 | |  | | |  | 1.0 | | |  | | 1.0 |  | |
| Intermediate risk (*n* = 488) | **1.94 (1.4-2.6)** | **<0.001** | 0.76 (0-2.2) | | 0.621 | | |  | **2.14 (1.3-3.5)** | | | **0.002** | | 1.11 (0.2-6.1) | 0.908 | |
| High risk (n = 7) | **9.32 (2.9-29)** | **<0.001** | 0 | | 0.952 | | |  | **21.6 (6.5-71)** | | | **<0.001** | | 0 | 0.966 | |
| Modified (n = 5) | 2.14 (0.3-15) | 0.448 | 0 | | 0.926 | | |  | 0 (0 - >100) | | | 0.964 | | 0 | 0.917 | |
| ^c^MRD x Final tx-interaktion | - | - | - | | n.s. | | |  | - | | | - | | - | n.s. | |
| ^c^Genetic subtype x Final tx-interaktion | - | - | - | | n.s. | | |  | - | | | - | | - | n.s. | |
| Genetic subtype |  |  |  | |  | | |  |  | | |  | |  |  | |
| High hyperdiploidy (*n* = 445) | 1.0 |  | 1.0 | |  | | |  | 1.0 | | |  | | 1.0 |  | |
| *ETV6*::*RUNX1* (*n* = 339) | 1.04 (0.6-1.7) | 0.882 | 0.92 (0.5-1.9) | | 0.882 | | |  | 0.97 (0.4-2.3) | | | 0.939 | | 1.32 (0.4-4.6) | 0.662 | |
| *TCF3*::*PBX1* (*n* = 47) | 0.69 (0.1-3.3) | 0.642 | 0.69 (0.1-3.3) | | 0.642 | | |  | 0.78 (0.1-6.0) | | | 0.811 | | 0.74 (0.1-7.0) | 0.794 | |
| dic(9;20)(p13;q11) (*n* = 27) | 1.32 (0.4-4.3) | 0.640 | 0 (0 - >100) | | 0.955 | | |  | 1.32 (0.2-10) | | | 0.788 | | 0 (0 - >100) | 0.956 | |
| iAMP21 (*n* = 23) | **5.37 (2.6-11)** | **<0.001** | **4.49 (1.9-11)** | | **<0.001** | | |  | **4.85 (1.4-17)** | | | **0.014** | | 2.53 (0.6-11) | 0.205 | |
| B-other (*n =* 340) | **2.71 (1.8-4.1)** | **<0.001** | **2.19 (1.2-4.0)** | | **0.010** | | |  | **4.46 (2.3-8.5)** | | | **<0.001** | | **2.88 (1.0-8.2)** | **0.048** | |
| Unknown (*n* = 60) | **2.90 (1.5-5.4)** | **0.001** | **2.70 (1.0-7.0)** | | **0.040** | | |  | 2.60 (0.8-8.0) | | | 0.098 | | 2.23 (0.4-12) | 0.352 | |
| White blood cell count x 10^9^/l |  |  |  | |  | | |  |  | | |  | |  |  | |
| <50 (*n* = 1125) versus ≥50 (*n* = 156) | 1.34 (0.9-2.1) | 0.174 | 1.35 (0.8-2.2) | | 0.212 | | |  | 1.37 (0.7-2.6) | | | 0.335 | | 1.32 (0.6-2.8) | 0.466 | |
| Age group (years) |  |  |  | | |  | |  |  | |  | | |  | |  |
| 1-9 (*n* = 966) | 1.0 |  | 1.0 | | |  | |  | 1.0 | | | |  | 1.0 | |  |
| 10-17 (*n* = 180) | **1.93 (1.3-2.9)** | **0.002** | 1.40 (0.9-2.2) | | | 0.139 | |  | **3.23 (1.7-6.0)** | | | | **<0.001** | **2.45 (1.4-4.8)** | | **0.009** |
| 18-45 (*n* = 135) | **3.90 (2.7-5.7)** | **<0.001** | **2.51 (1.6-3.9)** | | | **<0.001** | |  | **7.23 (4.2-12)** | | | | **<0.001** | **4.50 (2.3-8.9)** | | **<0.001** |
| CNS involvement |  |  |  | | |  | |  |  | | | |  |  | |  |
| CNS1 (*n* = 1150) | 1.0 |  | 1.0 | | |  | |  | 1.0 | | | |  | 1.0 | |  |
| CNS2 (*n* = 96) | 1.13 (0.7-2.0) | 0.678 | 1.41 (0.8-2.5) | | | 0.250 | |  | 1.98 (0.9-4.0) | | | | 0.056 | **2.82(1.4-5.9)** | | **0.006** |
| CNS3 (*n* = 32) | 1.01 (0.4-2.7) | 0.979 | 1.01 (0.3-3.2) | | | 0.985 | |  | 1.26 (0.3-5.2) | | | | 0.749 | 1.04 (0.2-6.8) | | 0.969 |
| Missing (*n* = 3) | 0 |  | 0 (0 - >100) | | | 0.899 | |  | 0 | | | |  | 0 (0 - >100) | | 0.882 |

BCP ALL, B-cell precursor acute lymphoblastic leukemia; CI, confidence interval; CNS, central nervous system; CNS1, no blasts on cytospin and no clinical

signs of CNS leukemia; CNS2, >0 and <5 cells/µl cerebrospinal fluid that on cytospin were regarded to represent leukemic blasts but no other signs of CNS leukemia; CNS3, ≥5 cells/µl cerebrospinal fluid that on cytospin were regarded to represent leukemic blasts and/or signs of CNS leukemia; Final tx, final treatment; HR, hazard ratio; iAMP21, intrachromosomal amplification of chromosome 21; *n*, number of patients; n.s., not significant.

Bold type indicates significance.

^a^BCP ALL patients stratified to SR (n=761, events=69, deaths=29), and IR (n=520, events=90, deaths=39).

^b^Final treatment administered is in some patients different from the intended treatment stratification according to the protocol (Supplementary Table X).

^c^MRD day 29 and the genetic subtype both influence the final treatment administered. Therefore, two multiplicative interaction variables, one for the interaction between MRD day 29 and final treatment and one for the genetic subtype and final treatment, are included in the multivariable models. These two interactions are presented as one merged variable each because none of the resulting multiplicative interaction variables in the model was significant.

**Supplementary Table 17** Frequencies of the genetic BCP ALL subtypes in relation to age

| Genetic subtype | Age group | | | *P* |
| --- | --- | --- | --- | --- |
|  | 1-9 years | 10-17 years | 18-45 years |  |
|  | *n* = 1071 | *n* = 227 | *n* = 195 |  |
| HeH (*n* = 471) | 402 (38%) | 48 (21%) | 21 (11%) | <0.001^a^ |
| *ETV6*::*RUNX1* (*n* = 346) | 326 (30%) | 17 (7.5%) | 3 (1.5%) |  |
| *KMT2A*-r (*n* = 51) | 26 (2.4%) | 8 (3.5%) | 17 (8.7%) |  |
| *TCF3*::*PBX1* (*n* = 49) | 33 (3.1%) | 9 (4.0%) | 7 (3.6%) |  |
| dic(9;20) (*n* = 29) | 26 (2.4%) | 1 (0.4%) | 2 (1.0%) |  |
| iAMP21 (*n* = 27) | 13 (1.2%) | 12 (5.3%) | 2 (1.0%) |  |
| HoL (*n* = 15) | 3 (0.3%) | 7 (3.1%) | 5 (2.6%) |  |
| NH (*n* = 8) | 8 (0.7%) | 0 | 0 |  |
| B-other (*n* = 421) | 201 (19%) | 111 (49%) | 109 (56%) |  |
| Unknown (*n* = 76) | 33 (3.1%) | 14 (6.2%) | 29 (15%) |  |

BCP ALL, B-cell precursor acute lymphoblastic leukemia; HeH, high hyperdiploidy (51-67

chromosomes); HoL, low hypodiploidy (30-39 chromosomes); iAMP21, intrachromosomal amplification of chromosome 21; *KMT2A*-r, *KMT2A* rearrangement; *n*, number of patients; NH, near-haploidy (24-29 chromosomes).

^a^Kruskal-Wallis one-way analysis of variance test.

**Supplementary Table 18** Frequencies of the final BCP ALL risk groups in relation to age

| Final risk | Age group | | |
| --- | --- | --- | --- |
| stratification | 1-9 years | 10-17 years | 18-45 years |
| group | *n* = 1071 | *n* = 227 | *n* = 195 |
| SR (*n* = 791) | 623 (58%) | 89 (39%) | 49 (25%) |
| IR (*n* = 490) | 343 (32%) | 91 (40%) | 86 (44%) |
| HR-chemo (*n* = 99) | 58 (5.4%) | 22 (9.7%) | 19 (9.7%) |
| HR-SCT (*n* = 95) | 34 (3.2%) | 23 (10%) | 38 (19%) |
| Not stratified (*n* = 18)^a^ | 13 (1.2) | 2 (0.9%) | 3 (1.5%) |

BCP ALL, B-cell precursor acute lymphoblastic leukemia; HR-chemo, high-risk patients stratified to

treatment with chemotherapy only; HR-SCT, high-risk patients stratified to stem cell transplantation; IR, intermediate risk; *n*, number of patients; SR, standard risk.

^a^These patients could not be assigned a final risk group because of induction death (*n* = 14), being lost to follow-up (*n* = 1), leaving protocol therapy before end of induction (*n* = 1), or having received such a modified therapy that they were considered outliers (*n* = 2).

**Supplementary Table 19** 5-year survival of the genetic BCP ALL subtypes in relation to age

| Genetic subtype | | Age group | | | | | *P* |
| --- | --- | --- | --- | --- | --- | --- | --- |
|  | 1-9 years | | | 10-17 years | | 18-45 years |  |
|  | *n* = 1071 | | | *n* = 227 | | *n* = 195 |  |
| HeH (*n* = 471) | | | *n* = 402 | | *n* = 48 | *n* = 21 |  |
| pCIR (no. of relapses) ± SE | | | 0.05 ± 0.01 (18) | | 0.08 ± 0.04 (4) | 0.12 ± 0.08 (2) | 0.055^a^ |
| pDFS (no. of events) ± SE | | | 0.93 ± 0.01 (28) | | 0.92 ± 0.04 (4) | 0.82 ± 0.09 (3) | 0.117^b^ |
| pEFS (no. of events) ± SE | | | 0.92 ± 0.01 (32) | | 0.92 ± 0.04 (4) | 0.82 ± 0.09 (3) | 0.207^b^ |
| pOS (no. of deaths) ± SE | | | 0.96 ± 0.01 (14) | | 0.98 ± 0.02 (1) | 0.95 ± 0.05 (1) | 0.966^b^ |
|  | | |  | |  |  |  |
| *ETV6*::*RUNX1* (*n* = 346) | | | *n* = 326 | | *n* = 17 | *n* = 3 |  |
| pCIR (no. of relapses) ± SE | | | 0.05 ± 0.01 (16) | | 0.20 ± 0.10 (3) | 0.33 ± 0.27 (1) | NA |
| pDFS (no. of events) ± SE | | | 0.92 ± 0.02 (24) | | 0.80 ± 0.10 (3) | 0.67 ± 0.27 (1) | NA |
| pEFS (no. of events) ± SE | | | 0.92 ± 0.02 (25) | | 0.88 ± 0.08 (2) | 0.67 ± 0.27 (1) | NA |
| pOS (no. of deaths) ± SE | | | 0.98 ± 0.01 (8) | | 0.92 ± 0.07 (1) | 0.67 ± 0.27 (1) | NA |
|  | | |  | |  |  |  |
| *KMT2A*-r (*n* = 51) | | | *n* = 26 | | *n* = 8 | *n* = 17 |  |
| pCIR (no. of relapses) ± SE | | | 0.08 ± 0.06 (2) | | 0.12 ± 0.12 (1) | 0.40 ± 0.13 (6) | 0.042^a^ |
| pDFS (no. of events) ± SE | | | 0.83 ± 0.08 (4) | | 0.75 ± 0.15 (2) | 0.47 ± 0.13 (8) | 0.068^b^ |
| pEFS (no. of events) ± SE | | | 0.77 ± 0.08 (6) | | 0.75 ± 0.15 (2) | 0.41 ± 0.12 (10) | 0.062^b^ |
| pOS (no. of deaths) ± SE | | | 0.81 ± 0.08 (5) | | 0.88 ± 0.12 (1) | 0.41 ± 0.12 (10) | 0.016^b^ |
|  | | |  | |  |  |  |
| *TCF3*::*PBX1* (*n* = 49) | | | *n* = 33 | | *n* = 9 | *n* = 7 |  |
| pCIR (no. of relapses) ± SE | | | 0.03 ± 0.03 (1) | | 0 | 0 | NA |
| pDFS (no. of events) ± SE | | | 0.97 ± 0.03 (1) | | 0.89 ± 0.10 (1) | 1.0 | NA |
| pEFS (no. of events) ± SE | | | 0.97 ± 0.03 (1) | | 0.89 ± 0.10 (1) | 1.0 | NA |
| pOS (no. of deaths) ± SE | | | 1.0 (0) | | 0.89 ± 0.10 (1) | 1.0 | NA |
|  | | |  | |  |  |  |
| dic(9;20) (*n* = 29) | | | *n* = 26 | | *n* = 1 | *n* = 2 |  |
| pCIR (no. of relapses) ± SE | | | 0.15 ± 0.07 (4) | | 0 | 0 | NA |
| pDFS (no. of events) ± SE | | | 0.81 ± 0.08 (5) | | − | 1.0 | NA |
| pEFS (no. of events) ± SE | | | 0.81 ± 0.08 (5) | | 1.0 | 1.0 | NA |
| pOS (no. of deaths) ± SE | | | 0.96 ± 0.04 (1) | | 1.0 | 1.0 | NA |
|  | | |  | |  |  |  |
| iAMP21 (*n* = 27) | | | *n* = 13 | | *n* = 12 | *n* = 2 |  |
| pCIR (no. of relapses) ± SE | | | 0.40 ± 0.14 (5) | | 0.25 ± 0.12 (3) | 0 (0) | NA |
| pDFS (no. of events) ± SE | | | 0.60 ± 0.14 (5) | | 0.67 ± 0.14 (4) | 1.0 (0) | NA |
| pEFS (no. of events) ± SE | | | 0.60 ± 0.14 (5) | | 0.67 ± 0.14 (4) | 1.0 (0) | NA |
| pOS (no. of deaths) ± SE | | | 0.92 ± 0.08 (1) | | 0.92 ± 0.08 (1) | 1.0 (0) | NA |
|  | | |  | |  |  |  |
| HoL (*n* = 15) | | | *n* = 3 | | *n* = 7 | *n* = 5 |  |
| pCIR (no. of relapses) ± SE | | | 0.33 ± 0.27 (1) | | 0.14 ± 0.13 (1) | 0.40 ± 0.22 (2) | NA |
| pDFS (no. of events) ± SE | | | 0.67 ± 0.27 (1) | | 0.57 ± 0.19 (3) | 0.60 ± 0.22 (2) | NA |
| pEFS (no. of events) ± SE | | | 0.67 ± 0.27 (1) | | 0.57 ± 0.19 (3) | 0.60 ± 0.22 (2) | NA |
| pOS (no. of deaths) ± SE | | | 0.67 ± 0.27 (1) | | 0.57 ± 0.19 (3) | 0.80 ± 0.18 (1) | NA |
|  | | |  | |  |  |  |
| NH (*n* = 8) | | | *n* = 8 | | *n* = 0 | *n* = 0 |  |
| pCIR (no. of relapses) ± SE | | | 0.12 ± 0.12 (1) | | − | − | NA |
| pDFS (no. of events) ± SE | | | 0.50 ± 0.18 (4) | | − | − | NA |
| pEFS (no. of events) ± SE | | | 0.50 ± 0.18 (4) | | − | − | NA |
| pOS (no. of deaths) ± SE | | | 0.50 ± 0.18 (4) | | − | − | NA |
|  | | |  | |  |  |  |
| B-other (*n* = 421) | | | *n* = 201 | | *n* = 111 | *n* = 109 |  |
| pCIR (no. of relapses) ± SE | | | 0.12 ± 0.02 (23) | | 0.16 ± 0.04 (17) | 0.34 ± 0.05 (34) | <0.001^a^ |
| pDFS (no. of events) ± SE | | | 0.85 ± 0.03 (29) | | 0.80 ± 0.04 (21) | 0.62 ± 0.05 (38) | <0.001^b^ |
| pEFS (no. of events) ± SE | | | 0.84 ± 0.03 (31) | | 0.80 ± 0.04 (22) | 0.62 ± 0.05 (38) | <0.001^b^ |
| pOS (no. of deaths) ± SE | | | 0.92 ± 0.02 (16) | | 0.87 ± 0.03 (14) | 0.74 ± 0.04 (26) | <0.001^b^ |
|  | | |  | |  |  |  |
| Unknown (*n* = 76) | | | *n* = 33 | | *n* = 14 | *n* = 29 |  |
| pCIR (no. of relapses) ± SE | | | 0.13 ± 0.06 (4) | | 0.21 ± 0.11 (3) | 0.24 ± 0.08 (6) | 0.592^a^ |
| pDFS (no. of events) ± SE | | | 0.87 ± 0.06 (4) | | 0.71 ± 0.12 (4) | 0.73 ± 0.09 (7) | 0.299^b^ |
| pEFS (no. of events) ± SE | | | 0.82 ± 0.07 (6) | | 0.71 ± 0.12 (4) | 0.73 ± 0.09 (7) | 0.599^b^ |
| pOS (no. of deaths) ± SE | | | 0.94 ± 0.04 (2) | | 0.79 ± 0.11 (3) | 0.85 ± 0.07 (4) | 0.314^b^ |

BCP ALL, B-cell precursor acute lymphoblastic leukemia; HeH, high hyperdiploidy (51-67

chromosomes); HoL, low hypodiploidy (30-39 chromosomes); iAMP21, intrachromosomal amplification of chromosome 21; *KMT2A*-r, *KMT2A* rearrangement; *n*, number of patients; NA, not applicable (too few deaths/events/relapses to allow statistically meaningful comparisons); NH, near-haploidy (24-29 chromosomes); pCIR, probability of cumulative incidence of relapse; pDFS, probability of disease-free survival; pEFS, probability of event-free survival; pOS, probability of overall survival; SE, standard error.

^a^Weighted log-rank test. ^b^Log-rank test.
